# Activation of E-prostanoid 3 receptor in macrophages facilitates cardiac healing after myocardial infarction

**DOI:** 10.1038/ncomms14656

**Published:** 2017-03-03

**Authors:** Juan Tang, Yujun Shen, Guilin Chen, Qiangyou Wan, Kai Wang, Jian Zhang, Jing Qin, Guizhu Liu, Shengkai Zuo, Bo Tao, Yu Yu, Junwen Wang, Michael Lazarus, Ying Yu

**Affiliations:** 1 Key Laboratory of Food Safety Research, CAS Center for Excellence in Molecular Cell Science, Institute for Nutritional Sciences, Shanghai Institutes for Biological Sciences, Chinese Academy of Sciences, University of Chinese Academy of Sciences, Shanghai 200031, China; 2 Department of Pharmacology, School of Basic Medical Sciences, Tianjin Medical University, Tianjin 300070, China; 3 Center for Genomic Sciences, LKS Faculty of Medicine, The University of Hong Kong, 5 Sassoon Road, Hong Kong, SAR 999077, China; 4 School of Life Science, Chinese University of Hong Kong, Hong Kong, SAR 999077, China; 5 Division of Biomedical Statistics and Informatics, Center for Individualized Medicine, Mayo Clinic, Scottsdale, Arizona 85259, USA; 6 Department of Biomedical Informatics, Arizona State University, Scottsdale, Arizona 85259, USA; 7 International Institute for Integrative Sleep Medicine (WPI-IIIS), University of Tsukuba, Tsukuba City, Ibaraki 305-8575, Japan

## Abstract

Two distinct monocyte (Mo)/macrophage (Mp) subsets (Ly6C^low^ and Ly6C^high^) orchestrate cardiac recovery process following myocardial infarction (MI). Prostaglandin (PG) E_2_ is involved in the Mo/Mp-mediated inflammatory response, however, the role of its receptors in Mos/Mps in cardiac healing remains to be determined. Here we show that pharmacological inhibition or gene ablation of the *Ep3* receptor in mice suppresses accumulation of Ly6C^low^ Mos/Mps in infarcted hearts. *Ep3* deletion in Mos/Mps markedly attenuates healing after MI by reducing neovascularization in peri-infarct zones. Ep3 deficiency diminishes CX3C chemokine receptor 1 (CX3CR1) expression and vascular endothelial growth factor (VEGF) secretion in Mos/Mps by suppressing TGFβ1 signalling and subsequently inhibits Ly6C^low^ Mos/Mps migration and angiogenesis. Targeted overexpression of Ep3 receptors in Mos/Mps improves wound healing by enhancing angiogenesis. Thus, the PGE_2_/Ep3 axis promotes cardiac healing after MI by activating reparative Ly6C^low^ Mos/Mps, indicating that Ep3 receptor activation may be a promising therapeutic target for acute MI.

## Abstract (web summary)

Acute myocardial infarction (AMI) triggers sterile inflammatory reaction mediated by prostaglandin E_2_ (PGE_2_). Tang *et al*. show that the PGE_2_ via its receptor EP3 promotes cardiac healing after AMI by recruiting reparative Ly6C_low_ monocytes/macrophages, which is mediated by TGF-β-driven regulation of CX3CR1 expression and VEGF secretion.

Healing of the infarcted myocardium involves a complex and coordinated process of inflammation, angiogenesis and tissue remodelling. Monocytes (Mos) and macrophages (Mps) in the infarcted myocardium are the essential immune cells for determining the progression and resolution of inflammation and repair following myocardial infarction (MI)^1^. Disruption of the Mo/Mp-mediated inflammatory response by Mo/Mp depletion or inhibition of Mo/Mp migration impairs wound healing and deteriorates left ventricular remodelling after MI^2^,^3^,^4^. In contrast, controlled activation of Mos/Mps ameliorates cardiac function and post MI remodelling by inducing Mo/Mp infiltration and neovascularization^5^. Two distinct subpopulations of Mos/Mps are involved in recovery after MI in a sequential pattern. Early responding Mos/Mps (Ly6C^high^) are predominant 1–3 days after MI and display phagocytic and pro-inflammatory properties highly expressing tumour necrosis factor (TNF) α, interleukin (IL-6), myeloperoxidase, and cathepsins, whereas late-responding Mos/Mps (Ly6C^low^) appear 4–7 days after MI and exhibit anti-inflammatory characteristics to support tissue regeneration by secreting reparative cytokines, such as IL-10, transforming growth factor (TGF) β1, and vascular endothelial growth factor (VEGF)^6^. Injured hearts express a unique chemokine profile over time to coordinate Mo recruitment, and the sequential recruitment of Ly6C^high^ and Ly6C^low^ Mos is dependent on C–C chemokine receptor type 2 (CCR2) and CX3C chemokine receptor 1 (CX3CR1), respectively^6^. Moreover, Ly6C^high^ Mos can convert to Ly6C^low^ Mos in circulation^7^,^8^ and inflamed tissues including infarcted hearts^9^,^10^. Delayed transition of Ly6C^high^ (M1-like) to Ly6C^low^ Mos/Mps (M2-like), such as in atherosclerotic or diabetic animals^11^,^12^, attenuates wound repair post MI. Thus, targeting certain Mos/Mps as a therapeutic strategy for infarct healing and repair continues to receive intensive attention^13^.

Prostaglandin (PG) E_2_ is a key lipid mediator in many pathophysiological processes, including inflammation and immune responses, and it elicits diverse functions by acting on its four different E-prostanoid receptors (Ep1–Ep4) in a paracrine and autocrine manner^14^. Microsomal PGE_2_ synthase-1 (mPGES-1), an inducible terminal isomerase for PGE_2_ biosynthesis, is the major source of PGE_2_ formed *in vivo* and during inflammatory responses^15^. Disabling PGE_2_ generation by mPGHS-1 deletion leads to increased infarct sizes and adverse left ventricular remodelling after MI in mice^16^. Interestingly, mice with mPGES-1 deletion in bone marrow-derived myeloid cells alone displayssimilar cardiac phenotypes in global mPGES-1-deficient mice as subjected to coronary ligation^17^, strongly implicating Mos/Mps-derived PGE_2_ in wound healing after MI. However, the underlying mechanisms are largely unknown.

In this study, we investigated the role of PGE_2_ receptors on recruitment of Mo/Mp subsets during inflammation and further explored effects of genetic deletion or overexpression of *Ep3* receptor in Mos/Mps on cardiac healing in mice after acute MI. We found, disruption of *Ep3* receptor in Mos/Mps resulted in augmented infarct size and reduced cardiac functions after MI through suppression of reparative Ly6C^low^ infiltration and its mediated angiogenesis in peri-infarct zones in mice. Moreover, *Ep3* deficiency in Mos/Mps inhibited TGFβ1 signalling to directly suppress transcription of *Cx3cr1* and *Vegf* genes, therefore, retarding Ly6C^low^ migration and neovascularization in the infarcted hearts. Controlled activation of *Ep3* receptor in Mos/Mps facilitated Ly6C^low^ infiltration and subsequent healing of MI by activation of TGFβ1 signalling. Thus, PGE_2_/Ep3 axis facilites cardiac healing after MI by activating reparative Ly6C^low^ Mos/Mps.

## Results

### Ep3 mediates recruitment of Ly6C^low^ Mos/Mps in peritonitis

**Figure 1:**
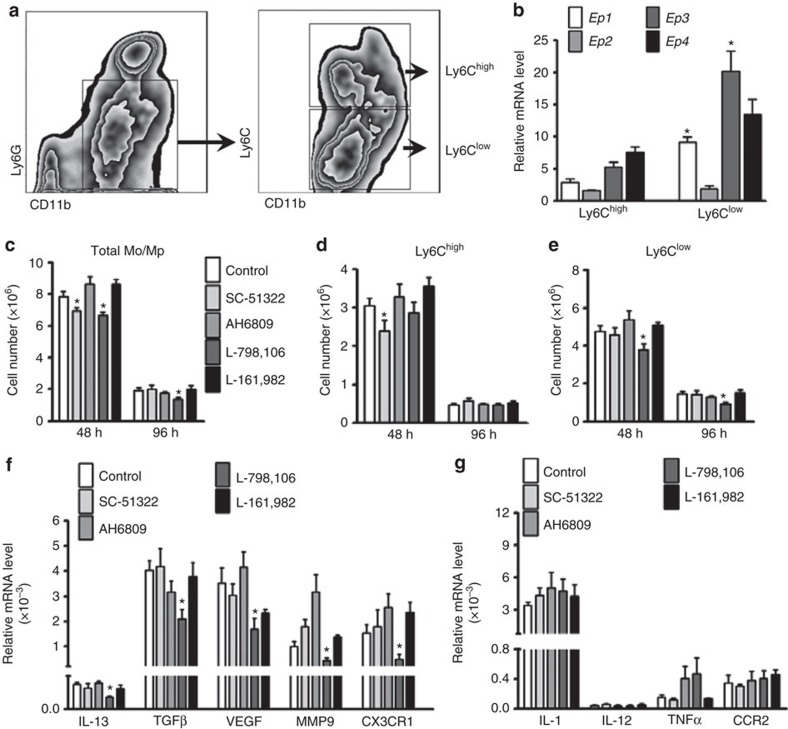
Ep3 blockade represses recruitment of Ly6C^low^ Mos/Mps in peritonitis in mice. (**a**) Gating strategy for peritoneal Ly6C^high^ and Ly6C^low^ Mos/Mps in zymosan-induced peritonitis in mice. (**b**) Relative mRNA levels of the PG E_2_ receptors (*Ep1–4*) in peritoneal Ly6C^high^ and Ly6C^low^ Mos/Mps; data represent mean±s.e.m. *P<0.05 versus Ly6C^high^Mos/Mps (unpaired two-tailed *t-*test); *n*=5. (**c**–**e**) Effect of administration of different PGE_2_ receptor blockers on recruitment of total Mos/Mps (**c**), Ly6C^high^ (**d**), and Ly6C^low^ (**e**) Mos/Mps at 48 h and 96 h after a zymosan challenge in mice. Data represent mean±s.e.m. *P<0.05 versus control(unpaired two-tailed *t*-test); *n*=5–6. SC-51322, *Ep1* inhibitor; AH6809, *Ep2* inhibitor; L-798106, *Ep3* inhibitor; L-161982, *Ep4* inhibitor. (**f**,**g**) Effect of different PGE_2_ receptor blockers on expression of anti-inflammatory (**f**) and pro-inflammatory (**g**) markers in peritoneal Mos/Mps collected 48 h after zymosan treatment. Data represent mean±s.e.m. *P<0.05 versus control (unpaired two-tailed *t*-test); *n*=4.

**Figure 2:**
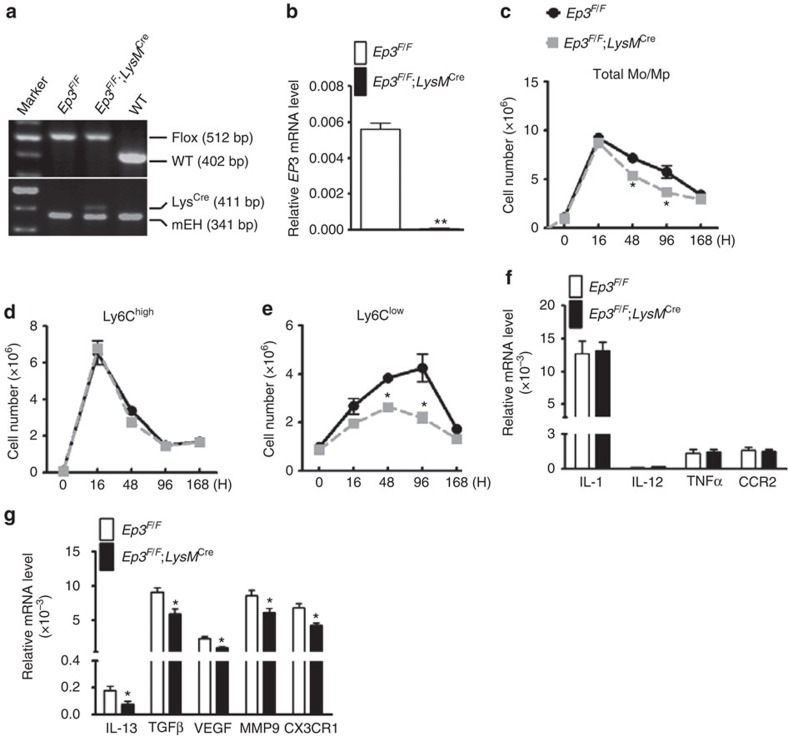
*Ep3* deletion suppresses Ly6C^low^ Mo/Mp infiltration in peritonitis in mice. (**a**) Genotyping of *Ep3*^*F/F*^*;LysM*^*Cre*^ mice. Microsomal epoxide hydrolase (mEH) was used as quality control for extracted DNA from mouse tail biopsy. (**b**) *Ep3* receptor expression levels in peritoneal Mps from *Ep3*^*F/F*^*;LysM*^*Cre*^ and *Ep3*^*F/F*^mice. Data represent mean±s.e.m. **P<0.01 versus *Ep3*^*F/F*^(unpaired two-tailed *t*-test); *n*=10. (**c**–**e**) Effect of *Ep3* deletion on recruitment of total Mos/Mps (**c**), Ly6C^high^ (**d**), and Ly6C^low^ subtype (**e**) Mos/Mps 48 and 96 h after a zymosan challenge in mice. Data represent mean±s.e.m. *P<0.05 versus *Ep3*^*F/F*^ (unpaired two-tailed *t*-test); *n*=5–7. (**f**,**g**) Effect of *Ep3* deficiency on expression of proinflammatory (**f**) and reparative angiogenic markers (**g**) in peritoneal Mos/Mps collected 48 h after zymosan treatment. Data represent mean±s.e.m. *P<0.05 versus *Ep3*^*F/F*^ (unpaired two-tailed *t*-test); *n*=10–11.

To explore which PGE_2_ receptor subtype(s) mediate Mo/Mp recruitment in zymosan-induced peritonitis in mice, both Ly6C^high^ and Ly6C^low^ Mos/Mps were sorted by flow cytometry (Fig. 1a). Western blot analysis confirmed that the surface markers CCR2 and CX3CR1 were abundantly expressed in Ly6C^high^ and Ly6C^low^ cells, respectively (Supplementary Fig. 1a), and reverse transcriptase polymerase chain reaction (RT-PCR) showed that more pro-inflammatory genes were expressed in Ly6C^high^ cells, while more reparative cytokines were expressed in Ly6C^low^ cells (Supplementary Fig. 1b,c). All PGE_2_ receptors (*Ep1–4*) were differentially expressed in both Ly6C^high^ and Ly6C^low^ Mos/Mps, and Ly6C^low^ Mos/Mps expressed more *Ep1* and *Ep3* receptors than Ly6C^high^ Mos/Mps (Fig. 1b). Interestingly, the *Ep3* receptor inhibitor L-798,106 reduced peritoneal infiltration of Ly6C^low^ and total Mos/Mps without significantly influencing Ly6C^high^ Mos/Mps, while the *Ep1* receptor inhibitor SC-51322 retarded recruitment of Ly6C^high^ Mos/Mps 48 h after the zymosan challenge (Fig. 1c–e). Consistently, L-798,106 significantly suppressed expression of reparative and pro-angiogenic cytokines (*IL-13, TGFβ1, VEGF* and *MMP9*) and *CX3CR1* in infiltrated Mos/Mps (Fig. 1f) without markedly altering expression of pro-inflammatory cytokines (Fig. 1g). In addition, by using myeloid cell-specific Ep3-deficient mice (*Ep3*^*F/F*^*;LysM*^*Cre*^, Fig. 2a), we also found Ep3 deficiency in Mos/Mps (Fig. 2b) markedly reduced total peritoneal Mos/Mps by restraining infiltration of Ly6C^low^ Mos/Mps in response to a zymosan challenge in mice (Fig. 2c–e). Similarly, *Ep3* deletion downregulated reparative cytokines and *CX3CR1* gene expression in Mos/Mps without overt effects on the expression of the pro-inflammatory cytokines tested (Fig. 2f,g).

We then examined the effect of *Ep3* deletion on the differentiation of recruited Mos in zymosan-induced peritonitis in mice. By day 1 after adoptive transfer (Supplementary Fig. 2a), ≈30% of the accumulated Ly6C^high^ Mos had Ly6C^low^ marker (Supplementary Fig. 2b), while *Ep3* deficiency did not significantly influence Ly6C^high^ Mo infiltration (Supplementary Fig. 2c) and its differentiation toward Ly6C^low^ Mos/Mps (Supplementary Fig. 2d). In contrast, deletion of *Ep3* receptor markedly reduced infiltration of Ly6C^low^ Mos/Mps in zymosan-challenged peritoneal cavity (Supplementary Fig. 2e,f). Taken together, these results suggest that the *Ep3* receptor is involved in mediating the recruitment of Ly6C^low^ Mos/Mps in response to inflammatory insults.

### *Ep3* promotes cardiac recovery by recruiting Ly6C^low^ Mo/Mp

**Figure 3:**
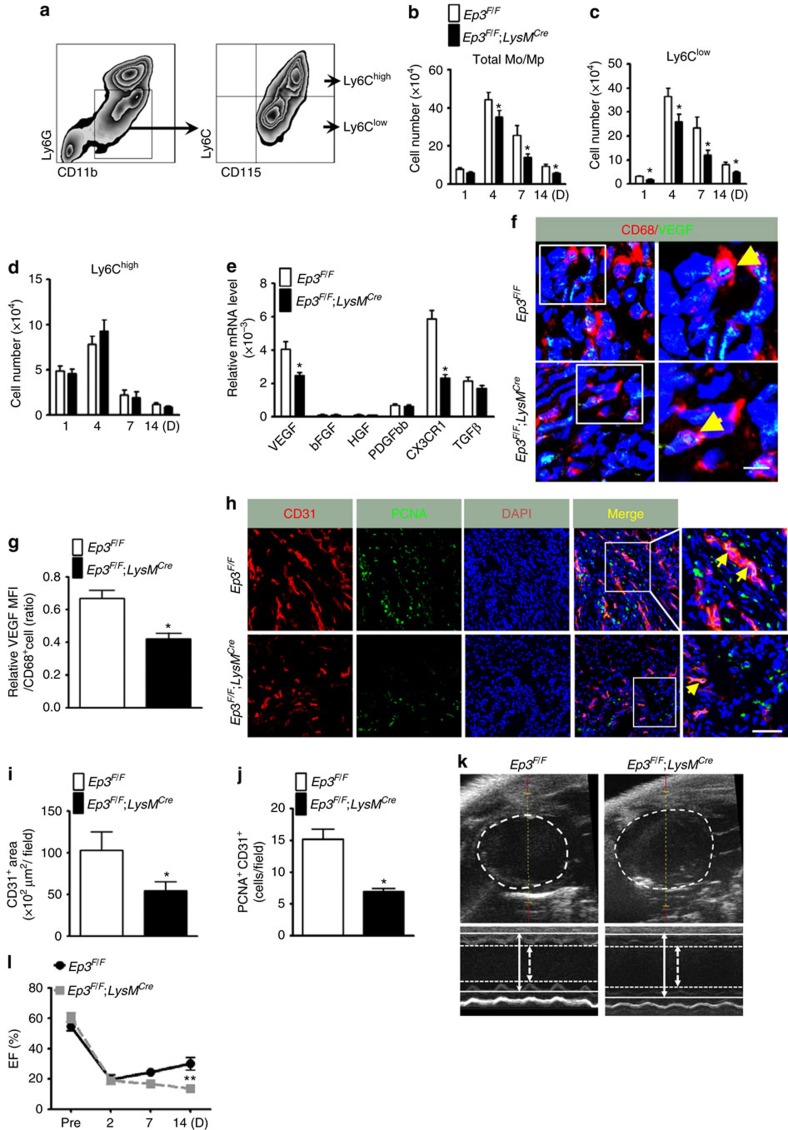
*Ep3* deletion retards cardiac recovery after MI in mice. (**a**) Gating strategy for CD11b^+^CD115^+^Ly6G^−^Ly6C^high^ and CD11b^+^CD115^+^Ly6G^−^Ly6C^low^ Mos/Mps in hearts after left anterior descending (LAD) artery ligation in mice. (**b**–**d**) Effect of *Ep3* deletion on recruitment of total Mos/Mps (**b**), Ly6C^low^ (**c**) and Ly6C^high^ (**d**) Mos/Mps in injured hearts of mice after MI. Data represent mean±s.e.m. *P<0.05 versus *Ep3*^*F/F*^ (unpaired two-tailed *t*-test); *n*=5–8. (**e**) mRNA expression levels of *VEGF*, fibrolast growth factor (*FGF*), hepatocyte growth factor (*HGF*), platelet-derived growth factor-bb (*PDGFbb*), and *CX3CR1* in Mos/Mps sorted from hearts in *Ep3*^*F/F*^ and *Ep3*^*F/F*^*;LysM*^*Cre*^ mice at day 14 post MI. Data represent mean±s.e.m. *P<0.05 versus *Ep3*^*F/F*^ (unpaired two-tailed *t*-test); *n*=10. (**f**) Representative immunostaining for CD68 (red) and VEGF (green) in peri-infarct zones of hearts from *Ep3*^*F/F*^and *Ep3*^*F/F*^*;LysM*^*Cre*^ mice at day 14 post MI. The solid box outlines the region enlarged to the right. Yellow arrow, CD68^+^/VEGF^+^ cell. Scale bar, 5 μm. (**g**) Quantitation of VEGF signalling in CD68^+^ cells in injured hearts as shown in **f**. Data represent mean±s.e.m. *P<0.05 versus *Ep3*^*F/F*^ (unpaired two-tailed *t*-test); *n*=5. (**h**) Representative immunostaining of CD31 (red) and proliferating cell nuclear antigen (PCNA, green) in peri-infarct zones of hearts from *Ep3*^*F/F*^ and *Ep3*^*F/F*^*;LysM*^*Cre*^ mice at day 14 post MI. The solid box outlines the region enlarged to the right; yellow arrow, CD31^+^/PCNA^+^ cell. Scale bar, 20 μm. (**i**,**j**) Quantitation of CD31^+^ areas (**i**) and PCNA^+^CD31^+^ cells (**j**) in injured hearts as shown in **h**. Data represent mean±s.e.m. *P<0.05 versus *Ep3*^*F/F*^ (unpaired two-tailed *t*-test); *n*=7. (**k**) Representative echocardiography images with M-mode views of infarcted hearts from *Ep3*^*F/F*^*;LysM*^*Cre*^ and *Ep3*^*F/F*^ mice on day 14 after MI. Arrows and lines mark left ventricular inner diameters (LVID) in systole (dashed) and diastole (firm). (**l**) Cardiac function of *Ep3*^*F/F*^*;LysM*^*Cre*^ and *Ep3*^*F/F*^ mice at different timepoints after MI. EF, ejection fraction. *P<0.05 versus *Ep3*^*F/F*^ (unpaired two-tailed *t*-test); *n*=9–13.

Two Mo/Mp subsets (Ly6C^low^ and Ly6C^high^) are also implicated in recovery after MI—a sterile inflammatory reaction^6^. We then examined the role of Mo/Mp in cardiac repair after ischaemia in mice. As expected, all PG products, including PGE_2_, were elevated in infarcted hearts, although *Ep3* deficiency in Mos/Mps had no significant influence on PG production (Supplementary Fig. 3a–e). CD11b^+^Ly6G^−^CD115^+^ Mos/Mps were sorted from infarcted hearts in mice (Fig. 3a). Notably, total infiltrated Mos/Mps in hearts were significantly reduced in *Ep3*^*F/F*^*;LysM*^*Cre*^ mice starting from 4 days after left anterior descending (LAD) artery ligation compared with those in EP3^F/F^ controls (Fig. 3b), through suppression of recruitment of CD11b^+^Ly6G^−^CD115^+^Ly6C^low^ Mos/Mps (Fig. 3c) not CD11b^+^Ly6G^−^CD115^+^Ly6C^high^ Mos/Mps (Fig. 3d), which was further confirmed by using additional F4/80 marker (Supplementary Fig. 4a–e). While myeloid-*Ep3* deficiency had no significant effect on total residential Mps in spleens, lungs, livers and hearts (Supplementary Fig. 5a–d), and on circulating Mos and neutrophils in mice either (Supplementary Fig. 6a,b). Again, Mo adoptive transfer confirmed *Ep3* deficiency resulted in decreased Ly6C^low^ infiltration without overt influence on Ly6C^high^ differentiation in circulation and infracted hearts (Supplementary Fig. 7a–i). The Ly6C^low^ Mo/Mp surface marker *CX3CR1* and *VEGF* expression in the infiltrated Mos/Mps were reduced by half in *Ep3*^*F/F*^*;LysM*^*Cre*^ mice (Fig. 3e), and the *CX3CR1* ligand *CX3CL1* expression was not altered in hearts from *Ep3*^*F/F*^*;LysM*^*Cre*^ mice (Supplementary Fig. 8a–c). Moreover, *Ep3* deletion did not influence proliferation and apoptosis of Mos/Mps infiltrated in infarcted hearts (Supplementary Fig. 9a–d). Immunostaining further confirmed reduction of VEGF expression in Mos/Mps in injured hearts in *Ep3*^*F/F*^*;LysM*^*Cre*^ mice (Fig. 3f,g). Accordingly, in *Ep3*^*F/F*^*;LysM*^*Cre*^ mice, neovascularization in the ischaemic zone at a late stage (day 14 after LAD ligation) was also diminished (Fig. 3h–j), infarct areas were expanded (Supplementary Fig. 10a,b), and cardiac function recovery was markedly impaired after MI (Fig. 3k,l, Supplementary Fig. 10c, Supplementary Table 1). However, *Ep3*^*F/F*^*;LysM*^*Cre*^mice had normal cardiac function at basal condition and even after dobutamine challenge (Supplementary Table 2), and *Ep3* deficiency did not influence the number and functions of neutrophil infiltrated in the infarcted hearts (Supplementary Fig. 11a–h).

Mps, as the major source of matrix metallopeptidases (MMPs) and TGFβ1, play an important role in cardiac remodelling and fibrosis^18^. Mo/Mp-Ep3 deletion, indeed, caused less non-vascular smooth muscle actin (SMA) positive myofibroblasts (Supplementary Fig. 12a,b), downregulation of *MMP9*, collagen I, III and Thrombospondin1 (*THBS1*) expression in infarcted hearts in mice (Supplementary Fig. 12c–j). Consistently, Masson's trichrome staining showed an increased myocardial scar size with decreased collagen deposition in *Ep3*^*F/F*^*;LysM*^*Cre*^ mice at both day 7 and 14 after MI (Supplementary Fig. 13a–c). We did not observe significant difference of early necrotic area between *Ep3*^*F/F*^*;LysM*^*Cre*^ and *Ep3*^*F/F*^ mice after MI (Supplementary Fig. 13d,e). Taken together, myeloid-Ep3 deletion impairs cardiac recovery from infarction by suppression of Ly6C^low^ Mo/Mp-mediated angiogenesis and cardiac fibrosis in mice (Supplementary Table 3).

Given that Ep3 deficiency inhibited *VEGF* expression in reparative Ly6C^low^ Mps, we tested the effect of the Mp Ep3 receptor on angiogenesis *in vitro*. Indeed, *VEGF* expression was diminished at both the mRNA (Supplementary Fig. 14a) and protein levels (Supplementary Fig. 14b,c) in cultured Mps from *Ep3*^*F/F*^*;LysM*^*Cre*^ mice. In a cultured three-dimensional angiogenesis model using HUVECs, their sprouting and tube structure formations were markedly reduced when co-cultured with peritoneal Mps from *Ep3*^*F/F*^*;LysM*^*Cre*^ mice compared to those in models co-cultured from *Ep3*^*F/F*^ controls (Supplementary Fig. 14d–g).

**Figure 4:**
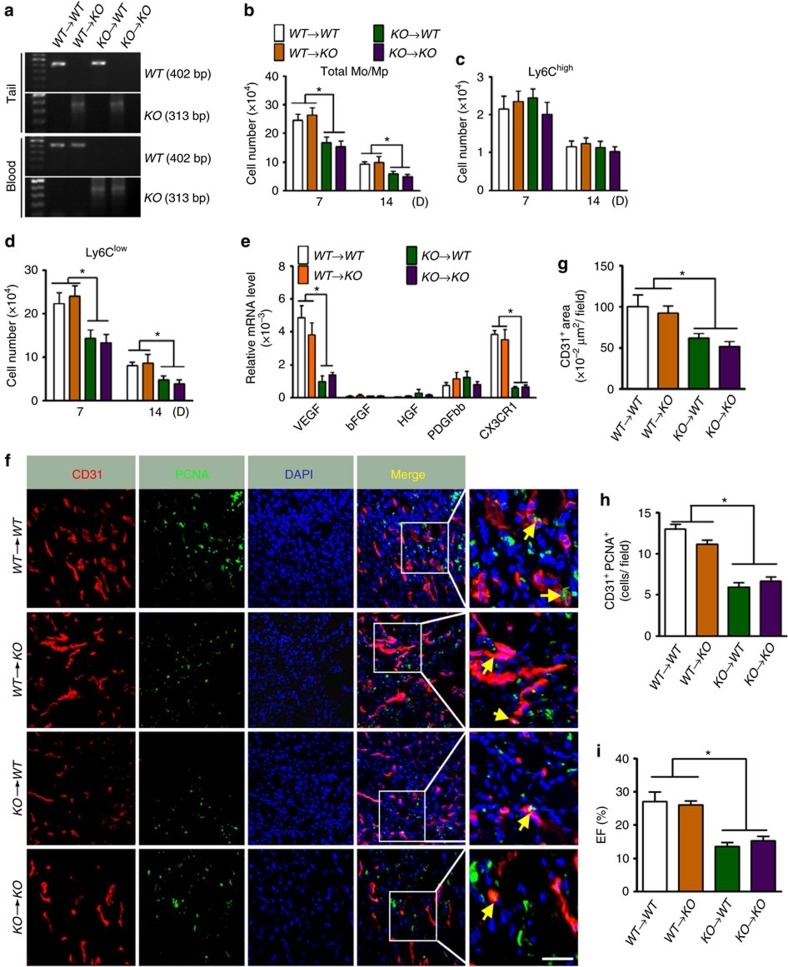
BMT ameliorates impaired cardiac function after MI in EP3 KO mice. (**a**) Bone marrow transplantation (BMT) between *Ep3* KO and WT mice was confirmed by genotyping. (**b**–**d**) Recruitment of total Mos/Mps (**b**), Ly6C^high^ (**c**) and Ly6C^low^ (**d**) Mos/Mps in injured hearts in chimeric mice that underwent BMT. Data represent mean±s.e.m. *P<0.05 as indicated (unpaired two-tailed *t*-test); *n*=6–7. (**e**) mRNA expression levels of *VEGF, FGF, HGF, PDGFbb* and *CX3CR1* in Mos/Mps sorted from hearts from BMT chimeric mice at day 14 post MI. Data represent mean±s.e.m. *P<0.05 as indicated (unpaired two-tailed *t*-test);*n*=4. (**f**) Representative immunostaining of CD31 (red) and PCNA (green) in peri-infarct zones of hearts from BMT chimeric mice at day 14 post MI. The solid box outlines the region enlarged to the right. Yellow arrow, CD31^+^/PCNA^+^ cells. Scale bar, 20 μm. (**g**,**h**) Quantitation of CD31^+^ areas (**g**) and PCNA^+^CD31^+^ cells (**h**) in injured hearts as shown in **f**. Data represent mean±s.e.m. *P<0.05 as indicated (unpaired two-tailed *t-*test); *n*=6–7. (**i**) Cardiac function of BMT chimeric mice at day 14 after MI. EF, ejection fraction. Data represent mean±s.e.m. *P<0.05 as indicated (unpaired two-tailed *t*-test); *n*=8–11.

To validate the role of Ep3 receptor in Mos/Mps, we examined whether bone marrow transplantation (BMT) from wild-type (WT) donors could rescue the defective function of Ly6C^low^ Mos/Mps in *Ep3* knockout (KO) mice. Genotyping of both tail specimens and blood samples verified successful BM reconstitution (Fig. 4a). Notably, total Ly6C^low^, not Ly6C^high^ Mos/Mps recruited in ischaemic hearts were significantly reduced in WT mice which underwent BMT from *Ep3* KO mice BM (KO→WT) (Fig. 4b–d). Moreover, the decreased expression of both *VEGF* and *CX3CR1* in *EP3* KO Ly6C^low^ Mos/Mps in the infarcted hearts was completely rectified by WT BMT (WT→KO, Fig. 4e). In line with the recovered infiltration and function of Ly6C^low^ Mos/Mps, neovascularization in peri-infarct zones and cardiac function after MI were significantly improved in WT→KO mice as compared with that in KO→KO mice (Fig. 4f–i, Supplementary Table 4).

### Ep3 deficiency inhibits CX3CR1 and VEGF expression in Mps

**Figure 5:**
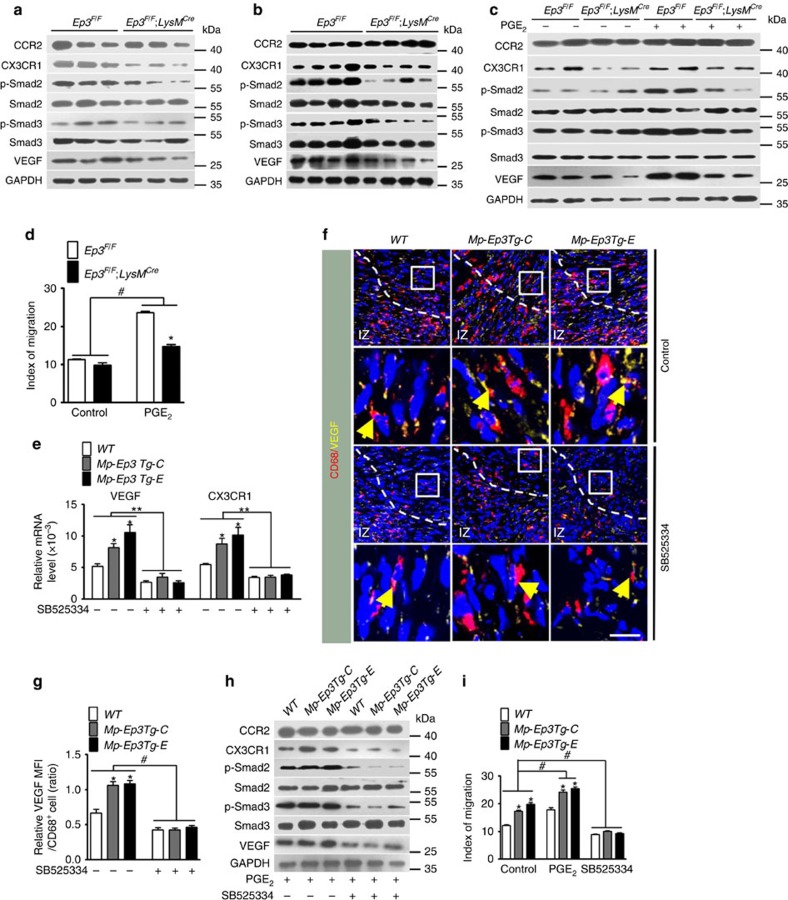
*Ep3* regulates CX3CR1 and VEGF expression through TGFβ1 signalling. (**a**,**b**)Western blot analysis of CCR2, CX3CR1, phospho-Smad2, phospho-Smad3 and VEGF in Mos/Mps from *Ep3*^*F/F*^ and *Ep3*^*F/F*^*;LysM*^*Cre*^ mice at 16 h (**a**) and 96 h (**b**)after a zymosan challenge. (**c**) Western blot analysis of CCR2, CX3CR1, phospho-Smad2, phospho-Smad3 and VEGF in cultured Mps with or without PGE_2_ stimulation. (**d**) Effect of *Ep3* deletion on Mp migration in response to PGE_2_. Data represent mean±s.e.m. *P<0.05 versus *Ep3*^*F/F*^, ^#^*P*<0.05 as indicated (unpaired two-tailed *t*-test); *n*=5. (**e**) Effect of TGFβ1 blocker SB525334 on Ep3-mediated *VEGF* and *CX3CR1* mRNA expression in cultured Mps. *Mp-Ep3Tg*, Mp-specific *Ep3α* transgenic mice. Data represent mean±s.e.m. *P<0.05 versus wild-type (WT), **P<0.01 versus indicated (unpaired two-tailed *t*-test); *n*=5. (**f**) Representative immunostaining of CD68 (red) and VEGF (green) in peri-infarct zones of hearts from *Mp-Ep3Tg* mice at day 14 post MI. The solid box outlines the region enlarged below. Yellow arrow, CD68^+^/VEGF^+^ cells. Scale bar 20 μm. IZ, infarct zone. (**g**) Quantitation of VEGF signalling in CD68^+^ cells in injured hearts as shown in **f**. Data represent mean±s.e.m. *P<0.05 versus WT, ^#^*P*<0.05 as indicated (unpaired two-tailed *t*-test); *n*=5. (**h**) Effect of SB525334 on *Ep3*-mediated VEGF and CX3CR1 protein expression in cultured Mps. (**i**) Effect of SB525334 on PGE_2_/*Ep3*-mediated Mp migration *in vitro*. Data represent mean±s.e.m. *P<0.05 versus WT, ^#^*P*<0.05 as indicated (unpaired two-tailed *t*-test); *n*=4–6.

Reparative Ly6C^low^ Mo/Mp recruitment during inflammation, including that post MI, depends on the *CX3CR1* receptor^6^. We detected striking downregulation of *CX3CR1* and *VEGF* expression in peritoneal Mps isolated from *Ep3*^*F/F*^*;LysM*^*Cre*^ mice challenged by zymosan at both 48 h (Fig. 5a, Supplementary Fig. 15a) and 96 h (Fig. 5b, Supplementary Fig. 15b) and in cultured *Ep3*-deficient Mps (*Ep3*^*F/F*^*;LysM*^*Cre*^ mice) treated by PGE_2_ (Fig. 5c, Supplementary Fig. 15c). CCR2, the receptor for monocyte chemoattractant protein-1 (MCP-1) that mediates pro-inflammatory Ly6C^high^ Mo/Mp recruitment, was not altered significantly in *Ep3*-deficient Mps (Fig. 5a–c). Accordingly, the migration of *Ep3*-deficient Mps in Boyden chambers was notably restrained in *Ep3*-deficient Mps with and without PGE_2_ stimulation (Fig. 5d).

**Figure 6:**
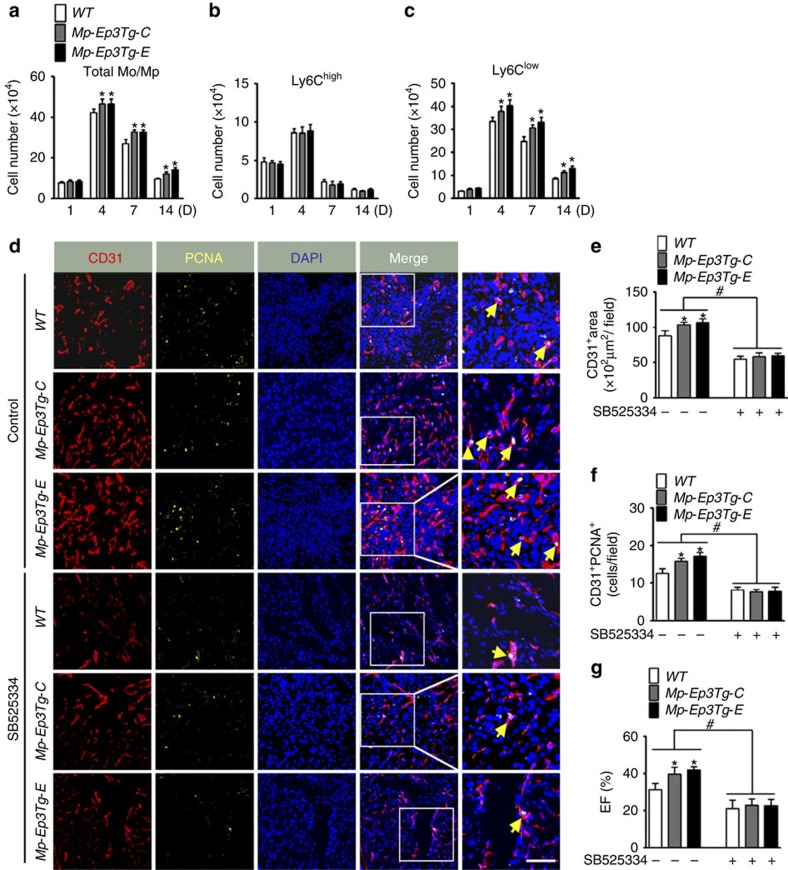
*Ep3* overexpression in Mos/Mps improves cardiac recovery after MI in mice. (**a**–**c**) Effect of *Ep3 o*verexpression on recruitment of total Mos/Mps (**c**), Ly6C^high^, (**d**) and Ly6C^low^ Mos/Mps in injured hearts mice after MI. Data represent mean±s.e.m. *P<0.05 versus WT (unpaired two-tailed *t*-test); *n*=5–7. (**d**) Representative immunostainings of CD31 (red) and PCNA (yellow) in peri-infarct zones of hearts from *WT, Mp-Ep3Tg-C* and *Mp-Ep3Tg-E* mice at day 14 post-MI. The solid box outlines the region enlarged to the right; yellow arrow, CD31^+^/PCNA^+^ cell. Scale bar, 20 μm. (**e**,**f**) Quantitation of CD31^+^ areas (**e**) and PCNA^+^CD31^+^ cells (**f**) in injured hearts, as shown in **d**. Data represent mean±s.e.m. *P<0.05 versus WT, ^#^*P*<0.05 as indicated(unpaired two-tailed *t*-test); *n*=6. (**g**) Cardiac function of *WT, Mp-Ep3Tg-C* and *Mp-Ep3Tg-E* mice at day 14 after MI. Data represent mean±s.e.m. *P<0.05 versus WT, ^#^*P*<0.05 as indicated (unpaired two-tailed *t*-test); *n*=7–8.

Previously, we demonstrated that the *Ep3* receptor mediates TGFβ1 signalling in pulmonary vascular smooth muscle cells by activation of Rho/ROCK^19^. Similarly, TGFβ1 signalling (phosphorylation of Smad2 and Smad3) was inhibited in *Ep3*-deficient Mps both *in vivo* and *in vitro* (Fig. 5a–c, Supplementary Fig. 15a–c). Additionally, *Ep3* deletion suppressed mouse Mp migration in response to PGE_2_ (Fig. 5d). In human blood CD14^dim^CD16^+^ Mos are similar to reparative Ly6C^low^ mouse Mos (ref. 20). *Ep3* agonist sulprostone promoted the migration of human CD14^dim^CD16^+^ Mos, while *Ep3* inhibitor L798106 suppressed CD14^dim^CD16^+^ Mo migration (Supplementary Fig. 16a,b). Likewise, *Ep3* receptor was also involved in regulation of *CX3CR1* expression in human Mos (Supplementary Fig. 16c). To investigate whether TGFβ1 signalling is involved in *Ep3*-mediated regulation of CX3CR1 and VEGF expression in Mos/Mps, we created an Mp-specific *Ep3α* transgenic (Mp-EP3Tg) mouse model using the CD68 promoter (Supplementary Fig. 17a–d). Indeed, overexpression of Mp-Ep3α induced activation of TGFβ1 signalling and elevated expression of both VEGF and CX3CR1 in Mps (Fig. 5e–h, Supplementary Fig. 18), and therefore increased PGE_2_-induced Mp migration (Fig. 5i), promoted infiltration of Ly6C^low^ Mos/Mps (Fig. 6a–c) and angiogenesis (Fig. 6d–f) in the infarcts, and facilitated cardiac recovery after MI (Fig. 6g). Interestingly, inhibition of TGFβ1 signalling markedly diminished the induction of VEGF and CX3CR1 expression (Fig. 5e–h) and augmented migration capacity *in vitro* in *Ep3α*-overexpressed Mps (Fig. 5i), retarded the increased Ly6C^low^ Mos/Mps accumulation in infarcts (Fig. 6c), and enhanced neovascularization in peri-infarct zones and cardiac function in *Mp-Ep3αTg* mice (Fig. 6d–g, Supplementary Table 5). We then tested whether the TGFβ1 pathway is implicated in *Mp-Ep3*-mediated angiogenesis using an Mp/HUVEC co-culture system. As shown in Supplementary Fig. 19a–c, forced expression of *Ep3α* in Mps increased VEGF expression at both the mRNA and protein levels, while the TGFβ1 signalling blocker SB525334 attenuated the elevated *VEGF* expression in *Mp-Ep3Tg* mice. Moreover, more sprouts and tubal structures from HUVECs containing beads were formed in culture with Mps from *Mp-Ep3Tg* mice than with those from WT controls (Supplementary Fig. 19d–g). Again, these differences were lost by the addition of SB525334 (Supplementary Fig. 19d–g). Thus, *Ep3*-mediated reparative Ly6C^low^ Mo/Mp recruitment and neovascularization in infarcted hearts is dependent on TGFβ1 signalling.

### CX3CR1 and VEGF are downstream targets of TGFβ1

**Figure 7:**
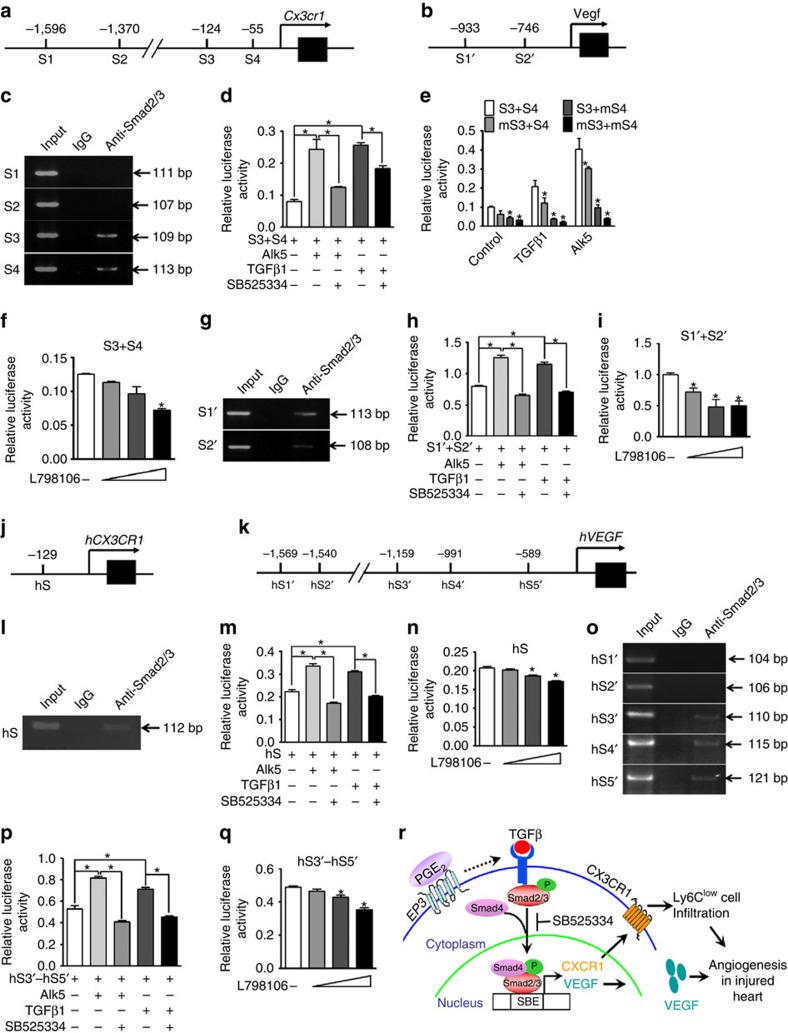
CX3CR1 and VEGF are TGFβ1-targeting genes in mice and humans. (**a**,**b**) Schematic illustration of predicted Smad binding elements (SBEs) in the promoter of murine *Cx3cr1* (**a**) and *Vegf* (**b**) genes. S=SBE. (**c**) Gel electrophoresis of PCR-amplified SBE-containing fragments in the promoter of the murine *Cx3cr1* gene using anti-Smad2/3 immunoprecipitation. (**d**) Effect of the TGFβ1 blocker SB525334 on S3+S4 fragment-mediated luciferase activity. *P<0.05 as indicated (unpaired two-tailed *t*-test); *n*=4. (**e**) Effect of mutations of S3 (mS3) and S4 (mS4) on S3+S4 fragment-mediated transcription activity. *P<0.05 versus the S3+S4 group, *n*=4. (**f**) Effect of L-798,106 on S3+S4 fragment-mediated luciferase activity in RAW264.7 cells. *P<0.05 versus no treatment (unpaired two-tailed *t*-test);*n*=4. (**g**) Gel electrophoresis of PCR-amplified SBE-containing fragments in the promoter of the murine *Vegf* gene using anti-Smad2/3 immunoprecipitation. (**h**) Effect of SB525334 on the functional SBEs (S1′+S2′) in murine *Vegf* promoter. *P<0.05 as indicated (unpaired two-tailed *t*-test); *n*=4. (**i**) Effect of L798106 on S1′+S2′ fragment-mediated luciferase activity in RAW264.7 cells. *P<0.05 versus no treatment (unpaired two-tailed *t*-test); *n*=5. (**j**,**k**) Schematic illustration of the predicted SBE(s) on the human *CX3CR1* gene (hS, (**j**)) and the human *VEGF* gene (hS′, (**k**)). (**l**) Gel electrophoresis of PCR-amplified SBE-containing fragments in the promoter of the human *CX3CR1* gene. (**m**) Effect of SB525334 on hS-mediated transcription activity in THP-1 cells. *P<0.05 as indicated (unpaired two-tailed *t*-test); *n*=5. (**n**) Effect of L-798,106 on hS-mediated transcription activity in THP-1 cells. *P<0.05 versus no treatment (unpaired two-tailed *t*-test); *n*=5. (**o**) Gel electrophoresis of PCR-amplified SBE-containing fragments in the promoter of the human *VEGF* gene. (**p**) Effect of SB525334 on hS3′–hS5′ fragment-mediated transcription activity in THP-1 cells. *P<0.05 as indicated, *n*=5. (**q**) Effect of L-798,106 on hS3′–hS5′ fragment-mediated transcription activity in THP-1 cells. *P<0.05 versus no treatment (unpaired two-tailed *t*-test); *n*=5. (**r**) Schematic diagram of EP3-mediated regulation of CX3CR1 and VEGF genes in Mos/Mps through TGFβ1.

Through the PWMScan tool, 4 and 2 potential Smad binding elements (SBEs) were predicted in the promoter of murine *Cx3cr1* (S1–4) and *Vegf* (S1′–2′) genes, respectively (Fig. 7a,b). However, the fragments containing S3 and S4 SBEs in the promoter of *Cx3cr1* were detected in the anti-Smad2/3 immunocomplex by Chip assay in mouse RAW264.7 cell line (Fig. 7c). Activation of TGFβ1 signalling by co-transfection of the TGFβ receptor Alk5 or addition of exogenous TGFβ1 markedly enhanced luciferase activities in the *Cx3cr1* promoter (S3+S4) containing reporter-transfected RAW264.7 cells, both of which could be specifically blocked by a TGFβ1 inhibitor (Fig. 7d). Mutation of either S3 or S4 significantly weakened the TGFβ1 and Alk5 co-transfection-induced luciferase activities in RAW264.7 cells as compared to these of WT fragment (S3+S4) transfected cells (Fig. 7e). Moreover, *Ep3* inhibition also suppressed S3+S4 promoter-driven activity in a dose-dependent manner (Fig. 7f). Similarly, two SBEs (S1′ and S2′) were identified in the *VEGF* promoter region by Chip and luciferase assays (Fig. 7g–i) in RAW264.7 cells. Moreover, 1 and 5 potential SBEs were predicted in the promoter of human *CX3CR1* (hS) and *VEGF* genes (hS1′–hS5′, Fig. 7j,k), respectively. Using a human THP-1 cell line, we identified one (hS, Fig. 7l–n) and three (hS3′–hS5′, Fig. 7o–q) functional SBEs in promoter of human *CX3CR1* and *VEGF* genes, respectively. Therefore, activation of the *EP3* receptor induces CX3CR1 and VEGF expression in Mps through TGFβ1 signalling and subsequently promotes Ly6C^low^ accumulation after MI and peri-infarct angiogenesis (Fig. 7r).

## Discussion

PGE_2_ is implicated in the regulation of multiple aspects of inflammation by tuning the functions of different immune cells, including Mos/Mps^21^. Mos/Mps are a dominant source of PGE_2_ at sites of inflammation^22^. In mice, Mos/Mps can be divided into two subsets based on the expression level of Ly6C (pro-inflammatory Ly6C^high^ and less inflammatory and patrolling Ly6C^low^)^6^. In humans, CD14^+^CD16^−^ Mos are similar to murine Ly6C^high^ cells, while CD14^dim^Mos are similar to murine Ly6C^low^Mos^20^. We found that *Ep3* mediates recruitment and reparative function of Ly6C^low^ Mo/Mp in inflamed tissues. Activation of the *Ep3* receptor upregulates expression of the Mp chemokine receptor CX3CR1, which mediates Ly6C^low^ Mo infiltration in hearts after MI^6^. In addition, the Mo/Mp *Ep3* receptor also mediates expression of the pro-angiogenic factor VEGF, which stimulates wound healing after MI by enhancing neovascularization in peri-infarct zones in mice. In agreement with our observations, tumour-derived PGE_2_ promotes differentiation of tumour-associated suppressive Mps (M2-like Mps) from the Mos infiltrated in tumours^23^. And the PGE_2_–*Ep3* axis also mediates recruitment and maturation of mast cells upon repeated allergen challenges^24^.

The Ep3 receptor mediates multiple cellular cascades by activating different types of heterotrimeric G proteins, including Gαs, Gαi and Gα12/13 (refs 25, 26). We recently discovered that activation of the Ep3 receptor augments Rho/ROCK-dependent TGFβ1/Smad2/3 signalling to facilitate hypoxia-induced vascular fibrosis by coupling with Gα12 in pulmonary arterial smooth muscle cells^19^. Indeed, Ep3 deficiency also results in decreased Rho activity and subsequent suppression of TGFβ1 signalling in both peritoneal and infarcted myocardium-derived Mps, whereas forced expression of the Ep3 receptor in Mos/Mps amplified TGFβ1/Smad2/3 signalling. Furthermore, we identified conservative SBEs in the promoter regions of both human and mouse *CX3CR1* genes coding a chemokine receptor for *CX3CL1*, which governs Ly6C^low^ Mo migration into inflammation sites^6^. Thus, the *Ep3* receptor regulates Ly6C^low^ Mo/Mp recruitment into the ischaemic myocardium through the TGFβ1/CX3CR1 pathway. Interestingly, *CX3CR1/CX3CL1* interactions confer a vital survival signal for Mos/Mps, whose complete disruption causes death of peripheral Mos^27^ and renal resident Mps^28^,^29^ and forms cells in atherosclerosis plaques^27^. We failed to observe marked death and/or apoptosis of peripheral Mos or Mps in the injured myocardium in *Mp-*Ep3-deficient mice with reduced expression of *CX3CR1* in Mos/Mps, which is consistent with the phenotypes presented in *CX3CR1* heterozygous mice (*CX3CR1*^*GFP/+*^)^27^,^30^. In addition, *CX3CR1* deficiency appears to impair reparative functions of macrophages (pro-fibrosis, proangiogenesis, and phagocytosis) in a variety of disease processes, such as skin wound healing^31^,^32^,^33^. In an experimental MI mouse model, Mo/Mp *Ep3* deletion retarded myocardial healing by inhibiting peri-infarct angiogenesis. Further mechanistic analysis revealed that Ep3 receptor activation in Mos/Mps promotes TGFβ1/Smad pathway-derived VEGF transcription. As previously reported^34^, we validated multiple conservative SBEs in the *VEGF* gene promoter in both mouse and human Mp cell lines, but not in infiltrated cardiac Mo/Mps due to technical difficulties. Taken together, the data suggest that activation of the Mo/Mp Ep3 receptor facilitates wound healing after MI by increasing CX3CR1-mediated Ly6C^low^ Mo recruitment and VEGF-induced angiogenesis, which are TGFβ1/Smad pathway-dependent.

Cardiac PGE_2_ generation increases during acute MI^17^. Despite the expression of multiple PGE_2_ receptor subtypes in hearts, selective stimulation of the Ep3 receptor displays cardio-protection against ischaemia/reperfusion injury in different mammalian species^35^,^36^,^37^,^38^. For instance, pharmacological activation of Ep3 receptor reduces myocardial infarct size in rodents by activation of protein kinase C (PKC) and the opening of ATP-sensitive potassium channels in cardiomyocytes^36^,^37^. We found that inactivation of *Ep3* in Mos/Mps impairs myocardial repair after acute ischaemia; in contrast, forced activation of *Ep3*-mediated signalling in Mos/Mps accelerates healing in mice after MI by increasing recruitment of reparative Mos/Mps and secretion of proangiogenic VEGF. Thus, Ep3 mediates cardiac protection against ischaemia, at least in part through activation of reparative Mos/Mps. In addition, Ep3 is also involved in regulation of lipid metabolism. Global *Ep3* deletion promotes to diet-induced obesity and exaggerates ectopic lipid deposition such as in skeletal muscle in mice^39^,^40^. Hepatic *Ep3* deficiency exacerbated atherosclerosis in hyperlidemic mice through suppression of biliary cholesterol secretion in mice^41^. However, Ep3 mediates vasoconstriction^42^, and genetic deletion of *Ep3* receptor increases bleeding tendency^38^ and reduces atherosclerosis-related thrombosis^43^, suggesting Ep3 involvement in platelet activation. Therefore, the potential efficacy of specific activation of Ep3 receptor in cardiac recovery after MI requires further investigations.

In summary, we showed that the PGE_2_–*Ep3* axis in Mo/Mp exerts a beneficial effect on myocardial recovery in response to acute ischaemia through TGFβ1-mediated activation of reparative Ly6C^low^ Mos/Mps. These observations suggest *Ep3* receptor and its downstream pathway maybe a promising therapeutic target for acute MI.

## Methods

### Mice

All animal procedures were approved by the Institutional Animal Care and Use Committee of the Institute for Nutritional Sciences, Chinese Academy of Sciences, as well as the number of animals to be used were approved based on the expected effects size. Ep3^F/F^ (*Ep3*^*F/F*^)^44^ and *Ep3* knockout (KO) mice^19^ were maintained on C57BL/6 background, *LysM*^*Cre*^ transgenic mice (C57BL/6, The Jackson Laboratory) were crossed *Ep3*^*F/F*^ to obtain *Ep3*^*F/F*^*;LysM*^*Cre*^ mice.

### Echocardiography

High-resolution echocardiography imaging system (Vevo 770, Visual Sonics) were used with M-mode analysis to assess mouse cardiac function. Both male and female mice (10–14 weeks old) were anaesthetized by isoflurane inhalation (1–2%) and heart rate was maintained at 350–500 b.p.m. The mitral valve leaflet was visualized and cardiac function was evaluated in the parasternal long axis view as previously described^45^.

### Macrophage-specific *Ep3α* transgenic mice

Macrophage (Mp)-specific *Ep3α* transgenic mice were generated as described previously^46^. Briefly, *Ep3*α cDNA was cloned into the CD68 promoter-contained vector, and the successful construct was injected into C57BL6 zygotes to obtain founder mice.

### Mouse model of myocardial infarction

Left anterior descending (LAD) ligation was used in both male and female mice (8–12 weeks old) to induce MI. Briefly, mice were anesthetized with isoflurane (2%) using an induction chamber, and the LAD coronary artery was completely ligated to induce left ventricular ischaemia^47^.

Myocardial necrotic injury was assessed by nitro blue tetrazolium (NBT) staining as previously reported^48^. Briefly, hearts were collected 12 or 24 h after LAD ligation and then cut into slices about 2 mm thick. Slices were incubated in 0.5 mg ml^−1^ NBT in phosphate buffer at pH 7.4 and 37 °C for 20 min. The unstainedportion (necrotic) could be separated from the dark blue stained (non-necrotic) region. The percentage of necrotic area was determined by dividing the weight of the necrotic tissue by the total weight and multiplying by 100.

As for infarction analysis, hearts were dissected at day 14 after infusion of 10% evans blue (100 μl) and frozen at −20 °C for 30 min, then cut into 2-mm-thick slices from apex to base. The slices were incubated in 1% triphenyltetrazolium chloride at 37 °C for 30 min to identify non-infarcted and infarcted areas. The normal myocardium was then recognized by red staining by triphenyltetrazolium chloride, while infarcted tissue presented as milky white. Infarct area was calculated as the ratio of the infarct area to the total slice areas previously described^49^.

### Enzyme-linked immunosorbent assay

Serum was collected from retro-orbital blood from mice 14 days after MI induction. Levels of the heart failure marker brain natriuretic protein in serum was measured by enzyme-linked immunosorbent assay, according to the manufacturer's instructions (R&D Systems).

### Immunofluorescence staining

For immunofluorescence, heart sections (8 μm) were incubated with primary antibodies against CD68 (Serotec, 1:200), CD301 (Biolegend, 1:200), PCNA (CST, 1:800), CD31 (RD, 1:200), VEGF (Proteintech, 1:200), SMA (Sigma, 1:500), CollagenΙ(Proteintech,1:200), THBS1(Proteintech, 1:200) and Alexa Fluor 488/594/633-conjugated secondary antibodies (Invitrogen, Carlsbad, CA, 1:1,000) for 1 h at room temperature, respectively. The embedded hearts were washed and stained with DAPI (Millipore, MA). Images were captured under a Olympus (FV1000) laser-scanning confocal microscope from each heart section for further analysis. Positive staining area and relative mean fluorescent intensity were measured using Image-Pro Plus software 6.0 (Media Cybernetics, Rockville, MD, USA)^50^.

For immunocytochemistry, primary Mos/Mps grown on slides were stimulated with or without PGE_2_ and then fixed with 4% paraformaldehyde. The slides were blocked in TBST containing 1% bovine serum albumin after washing, incubated with primary antibodies against CD68 (Serotec, 1:200) or VEGF (Proteintech, 1:200) overnight at 4 °C, stained with a secondary antibody for 2 h at room temperature, and photographed using fluorescent microscopy as described above. At least five random images were taken in region of each slide and positive signalling was quantified as previously described^51^.

### Bone marrow transplantation

Same sex BMT was performed as previously described^52^. In brief, mice (6–8 weeks old) were euthanized and bone marrow (BM) cells were collected from femurs and tibias. Recipient mice were lethally irradiated (one 5.0-Gy dose and another 4-Gy dose administered 1.5 h apart) from a 137Cs source (MDS Nordion, Ottawa, Ontario, Canada) and transplanted with 5 × 10^6^ donor BM cells via tail vein injection to reconstitute the hematopoietic system. Eight weeks after transplantation, BMT chimeric mice were used for experiments.

### Fluorescence activated cell sorting analysis

Cells collected from mice were stained with fluorochrome-conjugated antibodies, according to the manufacturer's protocols. FITC-conjugated antibodies to CD11b (M1/70,1:50), Brilliant Violet 421-conjugated antibodies to CD115 (AFS98,1:50), APC-conjugated antibody to Ly6C (HK1.4,1:50), APC-CY7-conjugated antibody to Ly6G (1A8,1:50) were purchased from Biolegend. Flow cytometry was performed using a BD FACS Aria flow cytometry system (BD Biosciences, San Jose, CA), and data were analysed with FlowJo software. Total viable leukocyte number was determined with the trypan-blue exclusion method. Leukocyte subpopulation numbers were calculated as total leukocytes multiplied by per cent cells within the selected population gated by FlowJo software (Tree Star, Ashland, Ore) and all FACS gates for fluorochrome-labelled monoclonal antibodies were defined using appropriate isotype controls.

### Peritoneal inflammation

Peritonitis was induced by intraperitoneal injection of zymosan (2 mg ml^−1^; Sigma-Aldrich, St Louis, MO). Peritoneal cells at different time points after the zymosan challenge were isolated for FACS analysis.

### Cell extract and culture

Primary Mos/Mps were prepared from the abdominal cavity and cultured in 10% FBS 1640 medium. A single-cell suspension from infracted hearts was prepared as previously described^53^. Briefly, the hearts were dissected, carefully cut into small pieces with fine scissors, and enzymatically digested with 450 U ml^−1^ of a cocktail of type I collagenase, 125 U ml^−1^ type IX collagenase, and 60 U ml^−1^ DNase I, and 60 U ml^−1^ hyaluronidase I-S (Sigma, St Louis, MO) for 1 h at 37 °C. After digestion, the tissues were passed through 70-μm cell strainers, washed, and stained with antibody for FACS analysis.

### Western blot

Cells were extracted in lysis buffer containing protease inhibitors. Protein concentration was determined by the BCA method using the Pierce BCA Protein Assay Kit (Pierce, Rockford, IL). Equal quantities of proteins from total cell lysates were separated by 10% SDS–PAGE gel and probed with antibodies against CX3CR1 (1:500, Abcam), CCR2 (1:500, Abcam), p-Smad2 (1:1,000, CST), p-Smad3 (1:1,000, CST), T-Smad3 (1:1,000, CST), T-Smad2/3 (1:1,000, CST), VEGF (1:1,000, Proteinteh), and GAPDH (1:2,000, CST), and then conjugated with an HRP-labelled secondary antibody in blocking buffer for 1–2 h at room temperature. Proteins were detected using an ECL detection kit (Super Signal West Pico Chemiluminescent Substrate), and signalling was quantified by image J software and presented as normalized arbitrary units. The uncropped scans of all western blots are supplied in Supplementary Fig. 20.

### Macrophage (Mp) migration assay

Primary Mps (1000,000 cells per well) were pretreated with 10 nM PGE_2_ in serum-free DMEM media for 24 h and then seeded on cell culture inserts (Millicell-PCF, Millipore) with porous polycarbonate filters (8-μm pore size) in 24-well plates. DMEM with CX3CL1 (20 ng ml^−1^) was placed in the lower chamber at 37 °C and 5% CO_2_. After 6 h, cells that migrated to the bottom well of the transwell chamber were fixed in paraformaldehyde (Thermo Scientific) and stained with DAPI for quantitation. The chemotactic index is the ratio of migrated Mps to total Mps.

### Quantitative RT-PCR analysis

RNA was isolated from tissues and cells using the Trizol reagent (Invitrogen, Carlsbad, CA), and cDNA was synthesized by reverse transcription kits (Takara, Dalian, China). All mRNA expression levels were normalized by comparing them to the housekeeping gene GAPDH. All RT-PCR primers are listed in Supplementary Table 6.

### Prediction of transcription-factor binding sites

The fragment between −2,000 and ±500 bp of the transcription start sites of the CX3CR1 and VEGF gene from the UCSC genome browser database was analysed for putative Smad binding element (SBE) motifs in TRANSFAC version 10.2 using PWMSCAN^54^. A match score with a *P* value <5 × 10^−6^ was considered to indicate a high-confidence binding site prediction.

### Chromatin immunoprecipitation (Chip) assays

Chip assays were conducted with a Magna ChIPTM A/G chromatin immunoprecipitation kit (Millipore) according to the manufacturer's protocol. In brief, RAW264.7 and THP-1 cells were stimulated for 24 h by TGFβ1. Cells were then cross-linked with formaldehyde (1% final concentration), and the pellet was lysed and sonicated to shear the chromatin into 200–1,000-bp fragments. The lysates were diluted using a chromatin dilution buffer. The chromatin extract was incubated with 10 μg of rat anti-Smad2/3 antibody (Abcam) or rat IgG (negative control) at 4 °C with rotation overnight, and the antibody–antigen–DNA complex was collected by protein G-agarose. The immunocomplexes were washed and the protein–DNA complexes were eluted, and then proteinase K was used to reverse the cross-linking of protein–DNA complexes to free up DNA. DNA was purified with the DNA purification kit (Promega), dissolved in elution buffer, and used for quantitative PCR analysis. The primers used for amplification of SBE-containing fragmentsof CX3CR1 and VEGF genes are listed in Supplementary Table 7.

### Luciferase reporter assay

Mouse macrophage cell line RAW264.7(ATCC TIB-71) and Human monocyte cell line THP-1(ATCC TIB-202) were tested every 3 months for mycoplasma contamination by performing a PCR on the cell supernatant. They were seeded into 24-well plates and grown to 70% confluence. Luciferase reporter or control empty vector plasmids were co-transfected with RPL-TK (internal plasmid) (20:1) into cells by using a lipofectamine 2000 transfection reagent (Invitrogen). Cells were cultured in a medium containing 1% FBS and lysed 24 h after transfection. The Ep3 antagonist L798106 (Cayman, 0.1–10 μmol) or TGFβ1 (Selleck, 10 ng ml^−1^) was used to treat cells overnight 16 h after transfection. Luciferase activities were monitored with a dual luciferase reporter assay kit (Promega).

### Plasmid construction

Mouse *Cx3cr1* promoter DNA fragments (−453 to 124 bp) and *Vegf* promoter DNA fragments (–1,216 to 399 bp) were amplified from mouse macrophage genomic DNA by PCR. The two promoter fragments were subcloned into pGL3 (Promega). The reporter with the insert of a fragment of −453 to 124 of *CX3CR1* gene was named S3+S4. Mutations were introduced into the third (mS3) and fourth (mS4) SBEs of the CX3CR1 promoter by QuikChange site-directed mutagenesis (Stratagene, La Jolla, CA). Human *CX3CR1* promoter DNA fragments (−196 to 107 bp) and VEGF promoter DNA fragments (−1,278 to 134 bp) were amplified and subcloned into the PGL3 vector for the reporter assay. The PCR primers for promoter subcloningare listed in Supplementary Table 8.

### *In vitro* HUVEC fibrin gel bead assay

Human vascular endothelial cells (HUVECs), from PromoCell (C-12200) were mixed with dextran-coated Cytodex 3 microcarriers (GE) at a concentration of 400 HUVECs per bead in 1.5 ml of EGM-2 medium (Clonetics, Walkersville, MD). Beads with cells were shaken gently at 37 °C and 5% CO_2_ every 20 min for 4 h. After incubating, beads with cells were transferred to a T2 tissue culture flask (Corning) and left overnight in 5 ml of EGM-2 at 37 °C and 5% CO_2_. After that, beads with cells were washed three times with 1 ml of EGM-2 and re-suspended at a concentration of 200 cell-coated beads per ml with 2.5 mg ml^−1^ of fibrinogen (Sigma) and 0.15 units ml^−1^ of aprotinin (Sigma). Five hundred microlitre of fibrinogen/bead solution was added to 0.625 units of thrombin (Sigma) in one well of a 24-well tissue culture plate. The fibrinogen/bead solution was allowed to clot for 5 min at room temperature and then at 37 °C and 5% CO_2_ for 20 min. One millilitre of EGM-2 (which contains 2% FBS) with or without 0.15 units ml^−1^ aprotinin was added to each well and equilibrated with the fibrin clot for 30 min at 37 °C and 5% CO_2_; 10^6^ macrophage cells were plated on top of the clot. Assays were terminated at day 7 for immunostaining and imaging^55^.

### PG extraction and analysis

Hearts collected from mice 14 days after MI were homogenized, and 500 μl supernatant were used for PG extraction. Heart prostanoid metabolites were extracted and quantitated using liquid chromatography/mass spectrometry/mass spectrometry (LC/MS/MS) analyses. In brief, following internal standards (2 μl), 40 μl citric (1M) and 5 μl BHT were added to the sample and then strenuously vibrated with 1 ml solvent (normal hexane: ethyl acetate, 1:1) for 1 min. After centrifugation (6,000 g min^−1^) for 10 min, the supernatant organic phase was collected and dried under a gentle stream of nitrogen, dissolved in 100 μl 10% acetonitrile in water. Production was normalized to total protein.

### Isolation of human blood Mos

Human peripheral blood mononuclear cells (PBMCs) were purchased from Zenbio and resuspended (10^6^ cells/10 μl) in sorting buffer (2 mM EDTA, 0.5% BSA in PBS) and stained with anti-human antibodies (Biolegend) specific for anti-CD14 and anti-CD16. Stained cells were filtered (70 μm) and sorted on a BD FACSAria II cell sorter using appropriate colour compensation for correcting spectral overlap and autofluorescence. Isolated CD14^dim^CD16^+^ Mos were used for subsequent experiments.

### Mo adoptive transfer

For zymosan-induced peritonitis mouse model, Mo subsets were sorted from spleens of EP3^F/F^ and EP3^F/F^Lys^Cre^ mice, then labelled with cell proliferation dye eFluor 670 (eBioscience) and injected into C57/BL6 mice 6 h after zymosan challenge by tail vain.

For MI model, Mo subsets were collected from CD45.2^+^ mice as previously described^10^, and injected into CD45.1^+^ mice on day 3 post MI. Infarcted hearts at day 6 post MI were dissected for preparation of single-cell suspension and flow cytometric analysis.

### Histological analysis

The infacted hearts were collected at indicated time points and embedded in the paraffin. The sections (6 μm) were stained with hematoxylin–eosin and Masson trichrome (Sigma). IPP software was used to analyse the necrotic area or collagen density as described^56^.

### Isolation of neutrophils and function assay

APC-conjugated antibody to CD11b (M1/70),PE-conjugated antibody to CD45(30-F11) and APC-CY7-conjugated antibody to Ly6G (1A8)were purchased from Biolegend. Neutrophils were sorted from injured hearts by FACS and calculated by FlowJo software (Tree Star, Ashland, Ore). Ly6G (ab25377) were purchased for immunofluorescence in mice heart after MI. For reactive oxygen species (ROS) measurement, purified neutrophils were loaded for 20 min at 37  °C with dichloro-dihydro-fluorescein diacetate (DCFH-DA). Cells were washed and then production of ROS was quantified via flow cytometry by measurement of DCF. To detect intracellular myeloperoxidase, 5 × 10^4^ neutrophils were lysed with TBS containing 0.2% Triton X-100 (50 ml) and the myeloperoxidase activity was measured as previously described^57^.

### Statistics

Prism software (GraphPad Prism version 5.0) was used for statistical analysis. Results are shown as mean±s.e.m. Two-tailed student's *t*-testing and one- or two-way ANOVA with Bonferroni *post-hoc* analyses were used for comparisons between different groups. A *P* value less than 0.05 was considered significant. Sample sizes were designed with adequate power according to the literature and our previous studies. Randomization and blinding strategy was used whenever possible.

### Data availability

All the data supporting the findings of this study are either included in the manuscript and its Supplementary Information Files, or can be obtained from the corresponding author upon reasonable request.

## Additional information

**How to cite this article:** Tang, J. *et al*. Activation of E-prostanoid 3 receptor in macrophages facilitates cardiac healing after myocardial infarction. *Nat. Commun.* **8,** 14656 doi: 10.1038/ncomms14656 (2017).

**Publisher's note**: Springer Nature remains neutral with regard to jurisdictional claims in published maps and institutional affiliations.

## Supplementary Material

Supplementary Information Supplementary Figures and Supplementary Tables.
