# Supplementary material for: Activation of E-prostanoid 3 receptor in macrophages facilitates cardiac healing after myocardial infarction
Source: Nat Commun. 2017 Mar 3;8:14656. doi: 10.1038/ncomms14656 (PMC5338035; doi:10.1038/ncomms14656)
Supplement: Supplementary Information — Supplementary Figures and Supplementary Tables. [file ncomms14656-s1.pdf]

Supplementary Figure 1

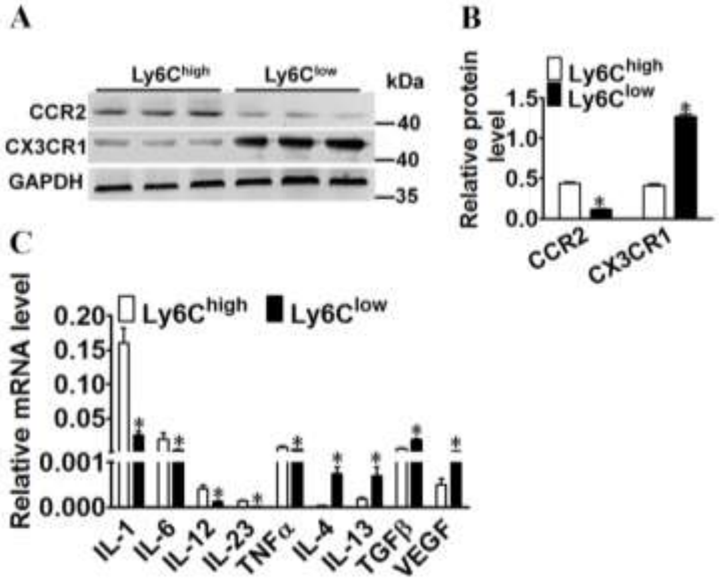

Supplementary Figure 1. Characterization of Ly6C^{high} and Ly6C^{low} Mos/Mps.
A. Western blot analysis of CCR2 and CX3CR1 expression in Ly6C^{high} and Ly6C^{low} Mos/Mps**.** Mos/Mps were sorted from the peritoneal cavity in zymosan-challenged mice. B. Densitometric quantification of *CCR2* and *CX3CR1* protein levels in Ly6C^{high} and Ly6C^{low} Mos/Mps. *P < 0.05 vs. Ly6C^{high}, n = 6. **C.** mRNA expression of pro-inflammatory and anti-inflammatory cytokines in Ly6C^{high} and Ly6C^{low} Mos/Mps. *P < 0.05 vs. Ly6C^{high}, n = 10.

Supplementary Figure 2

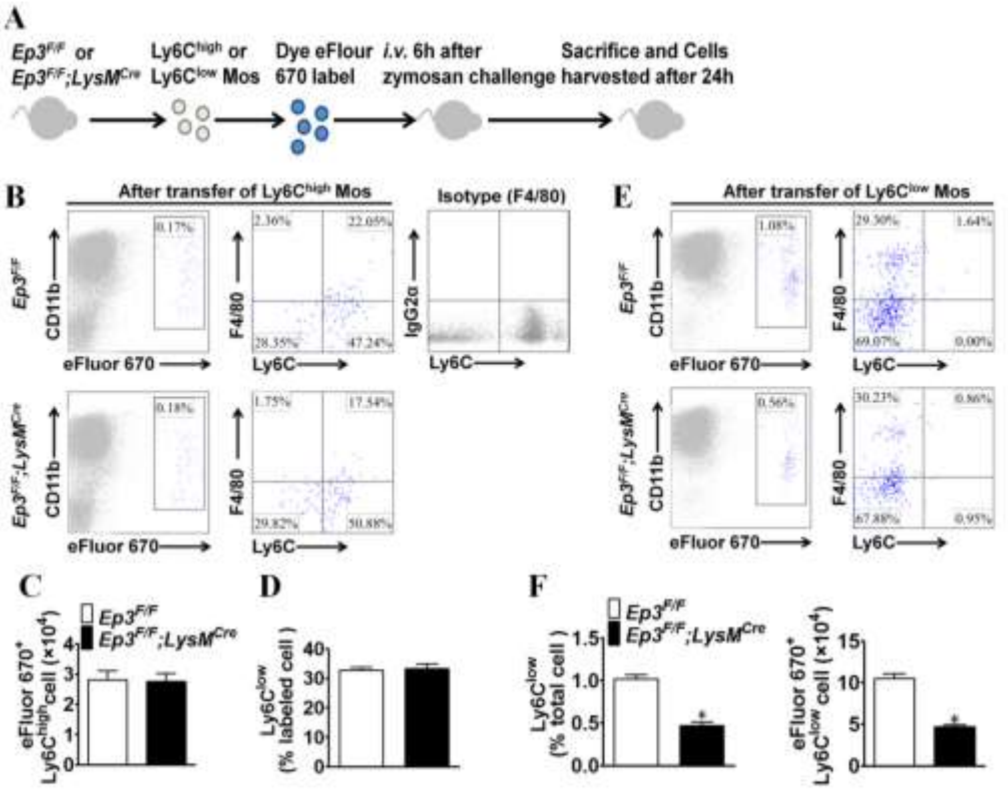

Supplementary Figure 2. Effect of *Ep3* deletion on peritoneal Mo differentiation in zymosan-challenged mice.

A. Illustration of experimental protocol with adoptive transfer of sorted Ly6C^{high} Mos (1×10^6) or Ly6C^{low} Mos (0.5×10^6) labeled with cell proliferation dye eFluor 670. **B.** Flow cytometric analysis of peritoneal eFluor 670$^+$Ly6C^{high} Mos in zymosan-challenged mice. **C**. Quantification of infiltrated eFluor 670$^+$Ly6C^{high} Mos/Mps as shown in **B.** n = 5-6. **D**. Quantification of differentiated eFluor 670$^+$Ly6C^{low} Mos/Mps as shown in **B**. n=5-6. **E.** Flow cytometric analysis of peritoneal eFluor 670$^+$Ly6C^{low} Mos in zymosan-challenged mice. **F**. Percentage (Left) and total number (Right) of infiltrated eFluor 670$^+$Ly6C^{low} Mos/Mps were calculated from **E**. *P < 0.05 vs. *Ep3*$^{F/F}$, n = 5-6.

Supplementary Figure 3

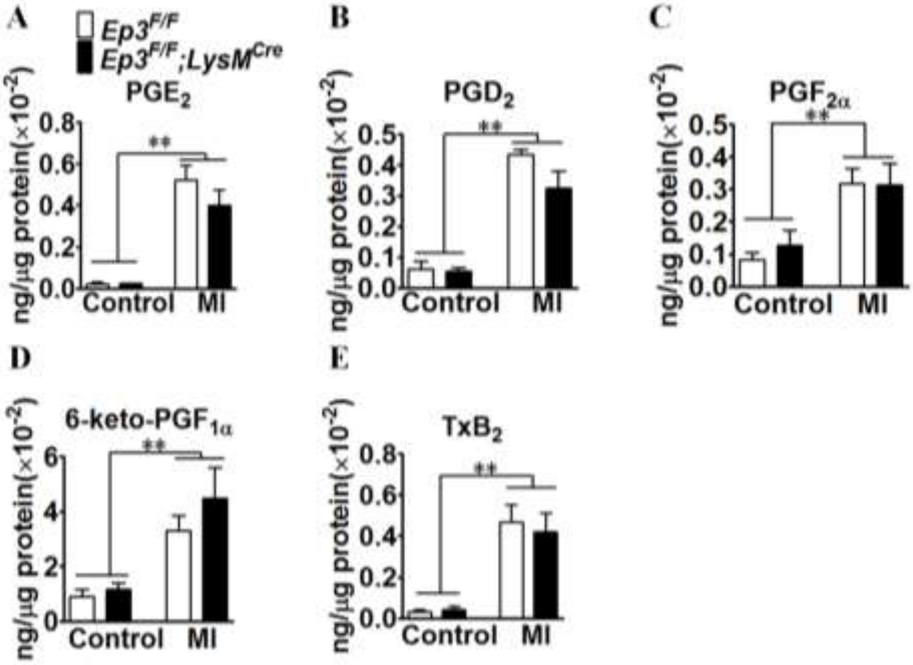

Supplementary Figure 3. PG production in ischemic hearts from *Ep3*$^{F/F}$ and *Ep3*$^{F/F}$*;LysM*^{Cre} mice after MI.

Mice underwent LAD ligation for 2 weeks, and then heart tissues were collected for PG extraction. **A-E**. PGE_2 **(A)**, PGD_2 **(B)**, $PGF_{2\alpha}$ **(C)**, 6-keto-$PGF_{1\alpha}$, a stable hydrolyzed product of PGI_2 **(D),** and TxB_2 **(E)** were examined by LC/MS/MS. **P < 0.01, n = 5.

Supplementary Figure 4

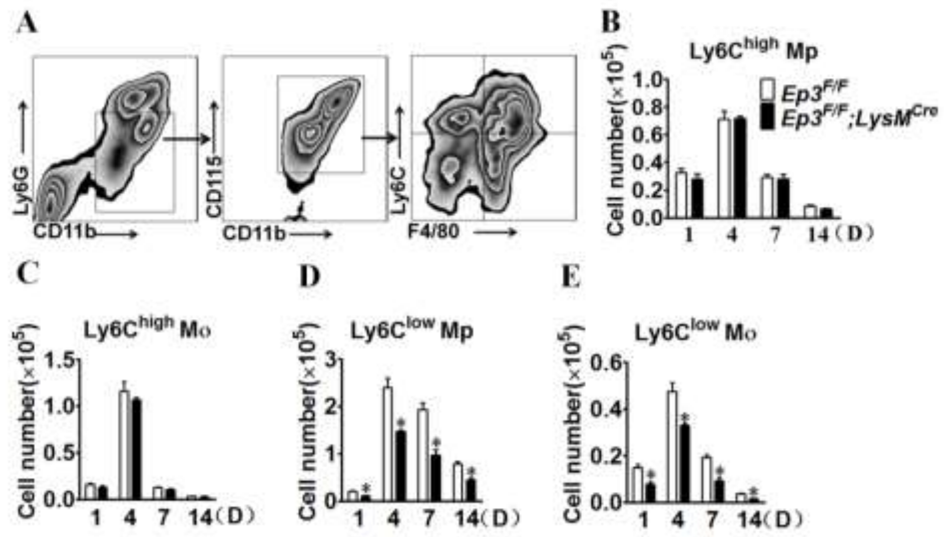

Supplementary Figure 4. Ep3 deletion reduces recruitment of Ly6C^{low}Mos /Mps.
A. Gating strategy for CD11b^{+}CD115^{+}Ly6G^{-}F4/80^{-}Ly6C^{high} Mos, CD11b^{+}CD115^{+} Ly6G^{-} F4/80^{-}Ly6C^{low} Mos, CD11b^{+}CD115^{+}Ly6G^{-}F4/80^{+}Ly6C^{high} Mps, CD11b^{+}CD115^{+} Ly6G^{-} F4/80^{+} Ly6C^{high} Mps in hearts after left anterior descending (LAD) artery ligation in mice. **B-D**. Effect of *Ep3* deletion on number of Ly6C^{high} Mps **(B)**, Ly6C^{high} Mos **(C),** Ly6C^{low} Mps **(D)** and Ly6C^{low} Mos **(E)** in injured hearts of mice after MI. *P < 0.05 vs. *Ep3$^{F/F}$*, n = 5-6.

Supplementary Figure 5

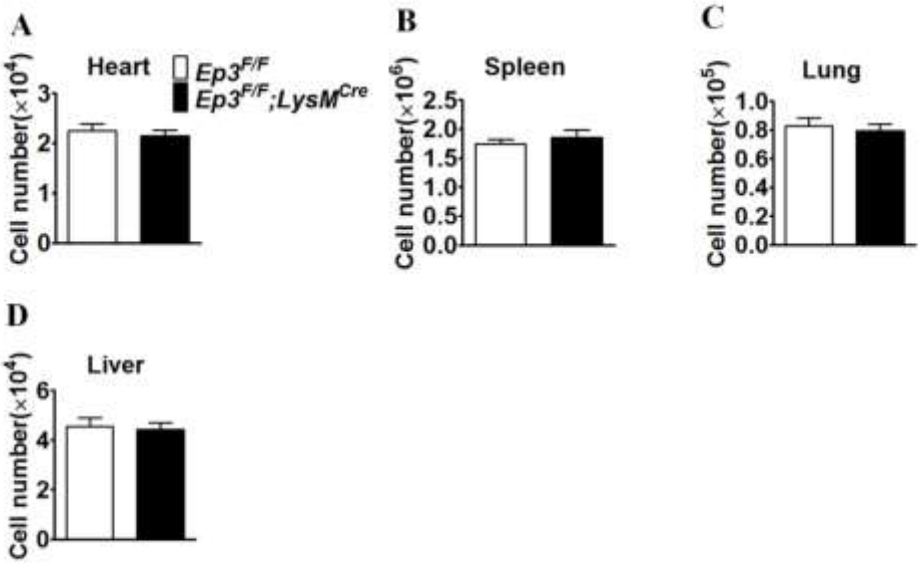

Supplementary Figure 5. Effect of myeloid-Ep3 deficiency on residential Mps in mice

Effect of *Ep3* deletion on total residential Mps in heart (A), spleen (B), lung (C), liver (D). n = 5-6.

Supplementary Figure 6

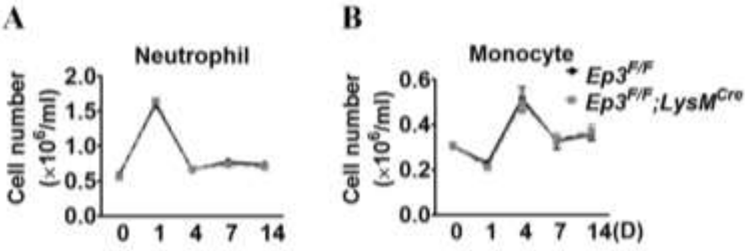

Supplementary Figure 6. Effect of myeloid-*Ep3* deficiency on circulating Mos and neutrophils in mice.

Quantification of plasma Mos (A) and neutrophils(B) in mice before and after MI at different timepoits. n =5.

Supplementary Figure 7

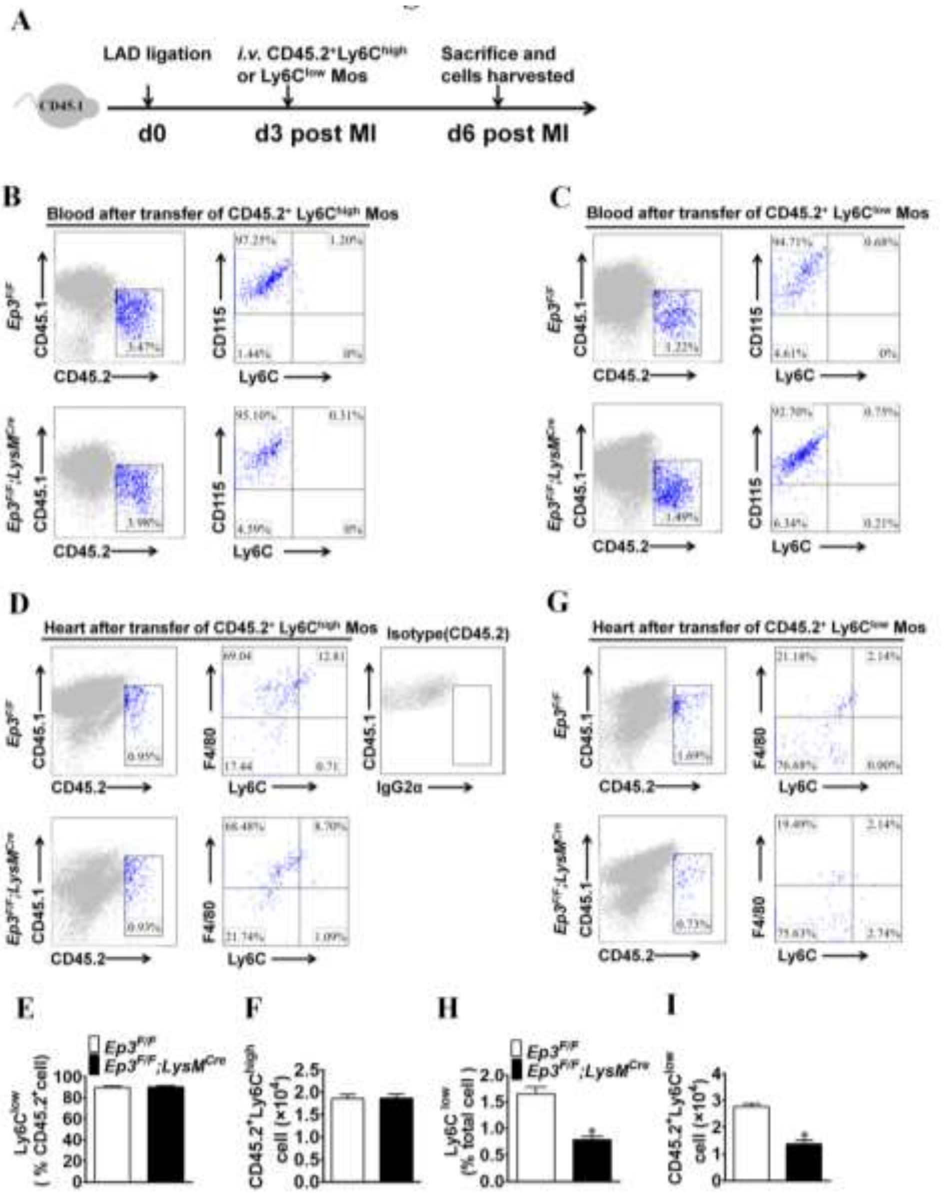

Supplementary Figure 7. Effect of *Ep3* deletion on Mo differentiation in infarcted hearts in mice.

A. Illustration of experimental approach with separate adoptive transfer of sorted $CD45.2^{+}Ly6C^{high}$ Mos (1×10^{6}) and $CD45.2^{+}Ly6C^{low}$ Mos (0.5×10^{6}) in $Ep3^{F/F};LysM^{Cre}$ and $Ep3^{F/F}$ ($CD45.1^{+}$) mice 3 days after MI. **B.** Flow cytometric analysis of blood $CD45.2^{+}$ $Ly6C^{high}$ Mos from $Ep3^{F/F};LysM^{Cre}$ and $Ep3^{F/F}$ mice. **C**. Flow cytometric analysis of blood $CD45.2^{+}Ly6C^{low}$ Mos from $Ep3^{F/F};LysM^{Cre}$ and $Ep3^{F/F}$ mice. **D.** Flow cytometric analysis of cardiac $CD45.2^{+}Ly6C^{high}$ Mos in $Ep3^{F/F};LysM^{Cre}$ and $Ep3^{F/F}$ mice after MI on day 3 after adoptive transfer. **E**. Quantification of differentiated $CD45.2^{+}Ly6C^{low}$ Mos/Mps as shown in **D**. n=4-5. **F**. Quantification of infiltrated $CD45.2^{+}Ly6C^{high}$ Mos/Mps as shown in **D**. n=4-5. n=4-5. **G.** Flow cytometric analysis of cardiac $CD45.2^{+}Ly6C^{low}$ Mos in $Ep3^{F/F};LysM^{Cre}$ and $Ep3^{F/F}$ mice after MI on day 3 after adoptive transfer. Percentage (**H**) and total number (**I**) of infiltrated $CD45.2^{+}Ly6C^{low}$ Mos/Mps were calculated from **G**. *$P < 0.05$ vs. $EP3^{F/F}$, n = 4-5.

Supplementary Figure 8

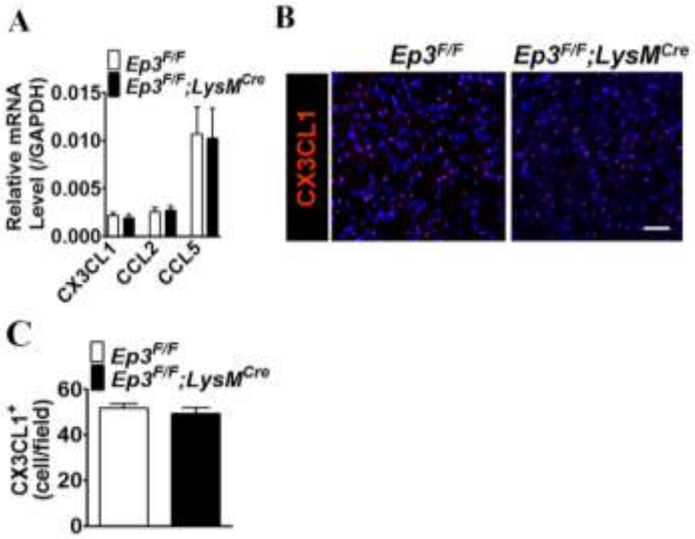

Supplementary Figure 8. Effect of *Ep3* deficiency on *CX3CR1* ligand in infarcted hearts in mice.

A. Relative mRNA levels of chemokine ligand *CX3CL1*, *CCL2* and *CCL5* in the infarcted hearts from *$EP3^{F/F}$;$LysM^{Cre}$* and *$Ep3^{F/F}$* mice at day 14 after MI. n=5. B. Representative CX3CL1 staining images of the infracted hearts from *$Ep3^{F/F}$;Lys^{Cre}* and *$Ep3^{F/F}$* mice at 14 after MI. n=5. Scale bar, 20 μm .C. Quantification of $CX3CL1^+$ cells in the infarcted hearts shown in B. n=5.

Supplementary Figure 9

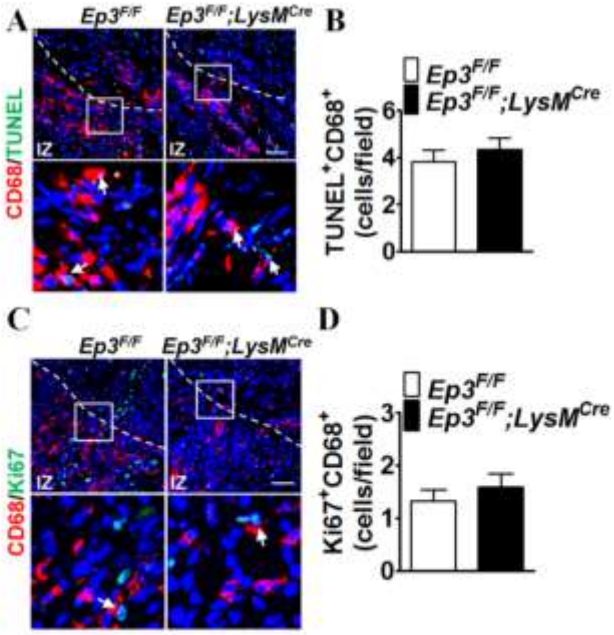

Supplementary Figure 9.Effect of *Ep3* deficiency in proliferation and apoptosis of Mps infiltrated in infarcted hearts.

A. Representative TUNEL$^+$CD68$^+$ cell staining images of the infracted hearts from *$Ep3^{F/F}$;$LysM^{Cre}$* and *$Ep3^{F/F}$* mice at day 7 after MI. Scale bar, 50μm. IZ, infarct zone. **B**. Quantification of TUNEL$^+$CD68$^+$ cells in infracted hearts at day 7 after MI. n=5. **C.** Representative Ki67$^+$CD68$^+$ cell staining images of infracted hearts from *$EP3^{F/F}$;$LysM^{Cre}$* and *$Ep3^{F/F}$* mice at day 7 after MI. Scale bar, 50 μm. **D**.Quantification of Ki67$^+$ CD68$^+$ cells in infracted hearts at day 7 after MI. n=5.

Supplementary Figure 10

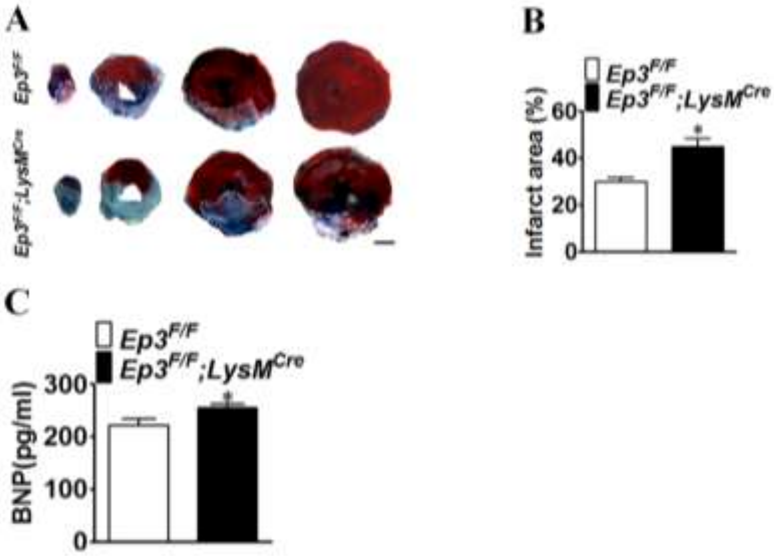

Supplementary Figure 10. ***Ep3*** **deficiency in Mos/Mps increases infarct size in mice after MI.**

A. Representative images of Evans blue and triphenyltetrazolium chloride staining on heart sections from $Ep3^{F/F}$ and $Ep3^{F/F};LysM^{Cre}$ mice two weeks after MI. The dotted line denotes the infarct zone. Scale bar, 1 ㎜. **B.** Infarct sizes were quantitated as shown in A. $^*P < 0.05$ vs. $Ep3^{F/F}$, n = 5. **C.** Brain natriuretic protein (BNP) was measured by enzyme-linked immunosorbent assay. $^*P < 0.05$ vs. $Ep3^{F/F}$, n = 10.

Supplementary Figure 11

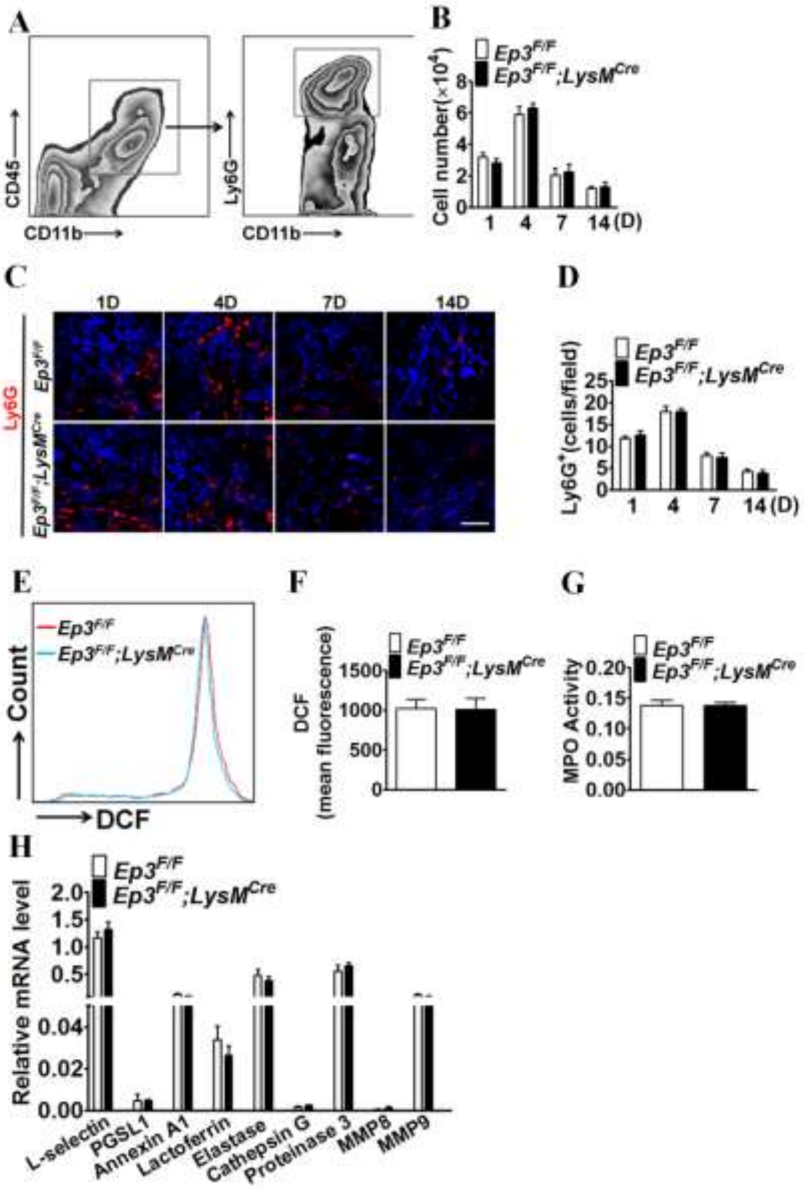

Supplementary Figure 11. Effect of *Ep3* deletion on neutrophil infiltration in hearts after MI.

A. Gating strategy for neutrophils in hearts from mice after MI. **B.** Effect of myeloid cell-Ep3 deletion on neutrophil infiltration in infarted hearts at different timepoits. n=6. **C**. Representative Ly6G$^+$ cell staining images of the infracted hearts from *Ep3$^{F/F}$;LysM^{Cre} and Ep3$^{F/F}$* mice after MI. Scale bar, 50 μm. **D.** Quantification of Ly6G$^+$ cells in infarcted hearts. n=5. **E-F** ROS levels in neutrophils isolated from infracted hearts at day 3 after MI. n=4. **G** MPO expression in neutrophils isolated from infracted hearts day 3 after MI. n=4. **H**. Relative mRNA levels of surface makers, proteases and matrix metallopeptidases of neutrophils isolated from infracted hearts at day 3 after MI. n=4.

Supplementary Figure 12

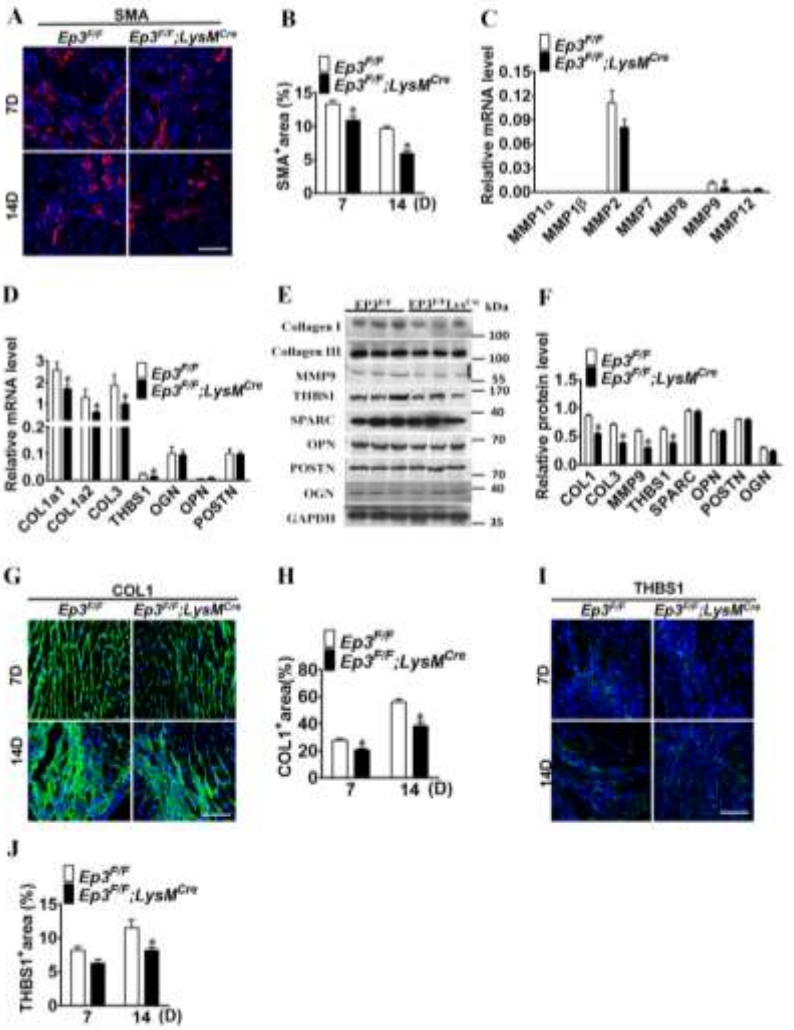

Supplementary Figure 12.Adverse cardiac remodelling in *$Ep3^{F/F};LysM^{Cre}$ mice* after MI

A. Representative SMA staining images of the infracted hearts from *$Ep3^{F/F};LysM^{Cre}$ and* *$Ep3^{F/F}$* mice at day 7 and 14 after MI. **B**. Quantification of SMA staining shown in **A**. *P < 0.05 vs. *$Ep3^{F/F}$*, n = 6. Scale bar, 50 µm. **C.** Relative mRNA levels of *MMPs* in the infracted hearts from *$Ep3^{F/F};LysM^{Cre}$* and *$Ep3^{F/F}$* mice at day 14 after MI. *P < 0.05 vs. *$Ep3^{F/F}$*, n = 6. **D**. Relative mRNA levels of fibrotic genes in the infracted hearts from $EP3^{F/F};LysM^{Cre}$ and *$Ep3^{F/F}$* mice at day 14 after MI. *P < 0.05 vs. *$Ep3^{F/F}$;*, n = 6. **E.** Western blot analysis of MMP9 and fibrotic proteins in the infracted hearts from *$Ep3^{F/F};LysM^{Cre}$* and *$Ep3^{F/F}$* mice at day 14 after MI. **F**. Quantification of MMP9 and fibrotic protein expression shown in **E.** *P < 0.05 vs. *$Ep3^{F/F}$*, n = 6. **G.** Representative collagen I staining images in the infracted hearts from *$Ep3^{F/F};LysM^{Cre}$* and *$Ep3^{F/F}$* mice at day 7 and 14 after MI. **H**. Quantification of collagen I staining shown in **G**. *P < 0.05 vs. *$Ep3^{F/F}$*, n = 6. Scale bar, 50 µm. **I.** Representative THBS1 staining images of the infracted hearts from *$Ep3^{F/F};LysM^{Cre}$* and *$Ep3^{F/F}$* mice at day 7 and 14 after MI. Scale bar, 50 µm. **J**. Quantification of THBS1 staining shown in **I**. *P < 0.05 vs. *$Ep3^{F/F}$*, n = 6.

Supplementary Figure 13

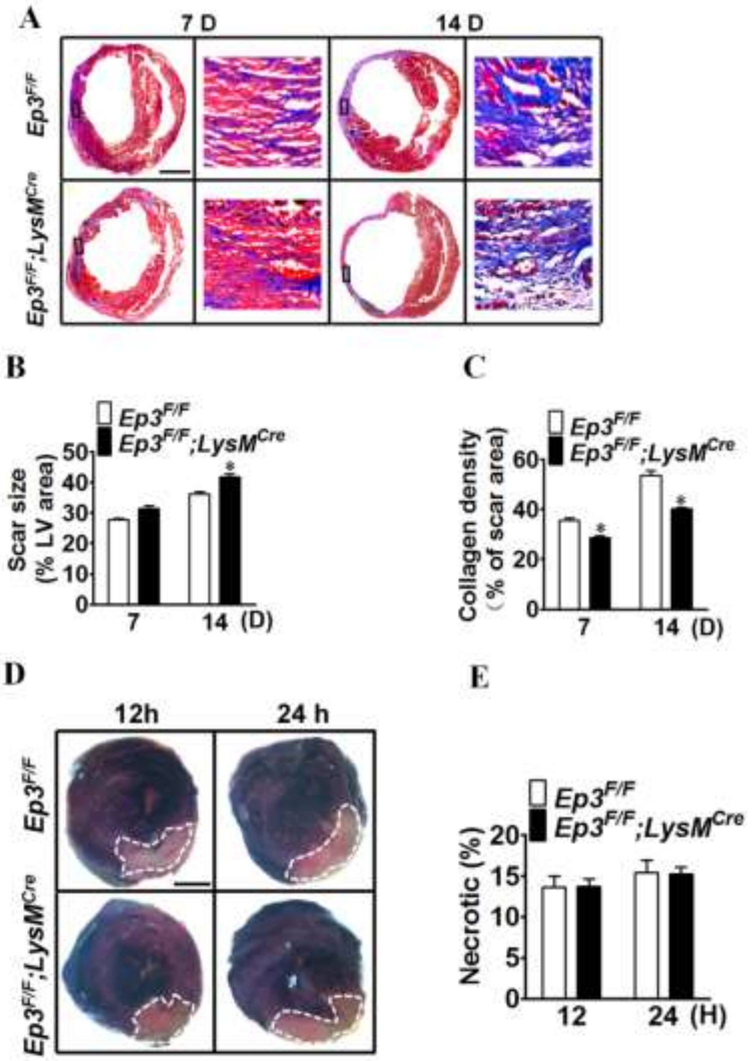

Supplementary Figure 13. Effect of myeloid-Ep3 deficiency on scar formation and necrosis in mice after MI.

A.Representative Masson's trichrome staining images of heart cross sections (2.0 mm distance from ligation site). from *$Ep3^{F/F}$;$LysM^{Cre}$* and *$Ep3^{F/F}$* mice at day 7 and 14 after MI. Scale bar, 1㎜. **B**. Quantification of collagen I density within scar area shown in **A**. *P < 0.05 vs. *$Ep3^{F/F}$*, n = 6. **C.** Quantification of scar size in infracted hearts shown in **A** . *P < 0.05 vs. *$Ep3^{F/F}$*, n = 6. **D.** Representative NBT staining images of heart sections (mid-papillary muscle level) from *$Ep3^{F/F}$;$LysM^{Cre}$* and *$Ep3^{F/F}$* mice 12 hours and 24 hours after LAD ligation. Scale bar, 1㎜. **E.** Quantification of necrotic areas in infracted hearts shown in **D** . n = 5.

Supplementary Figure 14

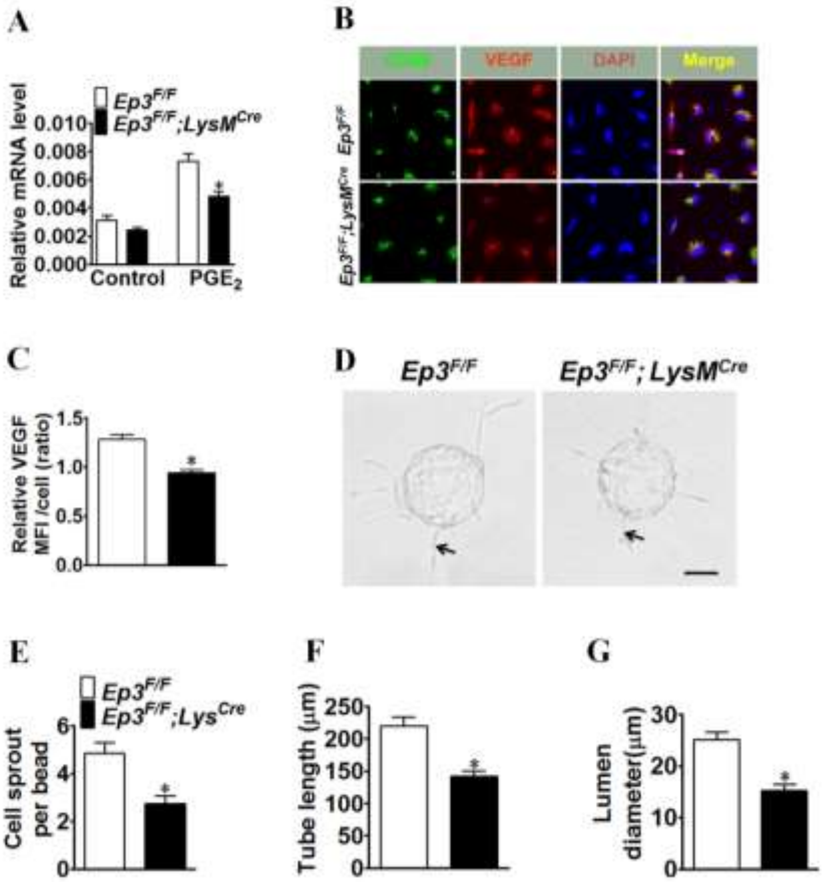

Supplementary Figure 14.Mp *Ep3* deficiency reduces VEGF secretion and tube formation of co-cultured human vascular endothelial cells (HUVECs) *in vitro*.

A. *VEGF* mRNA expression levels in peritoneal Mos/MPs from $Ep3^{F/F}$;$LysM^{Cre}$ and $Ep3^{F/F}$ mice. *P < 0.05 vs. $Ep3^{F/F}$, n =10. **B.** Representative immunostaining of CD68 (green) and VEGF (red) in peritoneal Mps from $Ep3^{F/F}$;$LysM^{Cre}$ and $Ep3^{F/F}$ mice. Scale bar, 20 µm. **C.** Quantitation of VEGF signaling in $CD68^+$ cells in Mos/MPs as shown in (**B**). *P < 0.05 vs. $Ep3^{F/F}$, n = 6. **D-G.** Fibrin bead-bound HUVECs were co-cultured with Mps from $Ep3^{F/F}$;$LysM^{Cre}$ and $Ep3^{F/F}$ mice, and multiple capillary-like sprouts were displayed on day 7 (**D**). Black arrow, capillary-like sprout; scale bar, 20 µm. The number of vessel sprouts per bead (**E**), the lumen diameter (**F**), and single-tube length per bead (**G**) were quantified at day 7. *P < 0.05 vs. $Ep3^{F/F}$, n = 4.

Supplementary Figure 15

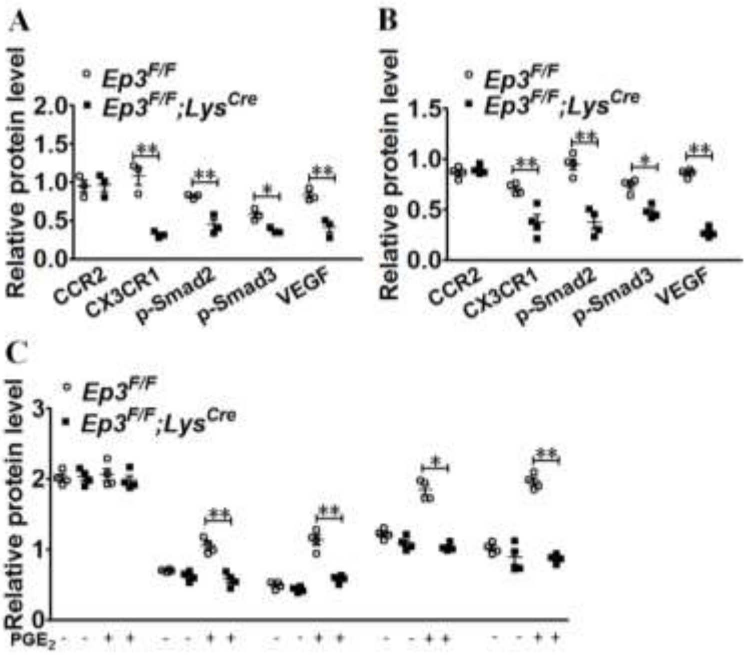

Supplementary Figure 15. Effect of *Ep3* deletion on expression of CCR2 and CX3CR1 and TGFβ1 signaling in Mps.

A-C. Densitometric quantification of CCR2, p-Smad2/3, CX3CR1, and VEGF protein expression of peritoneal Mps from in zymosan-challenged mice, as shown in Figure 5A-5C. *P < 0.05 vs. *Ep3*$^{F/F}$, n = 3-4.

Supplementary Figure 16

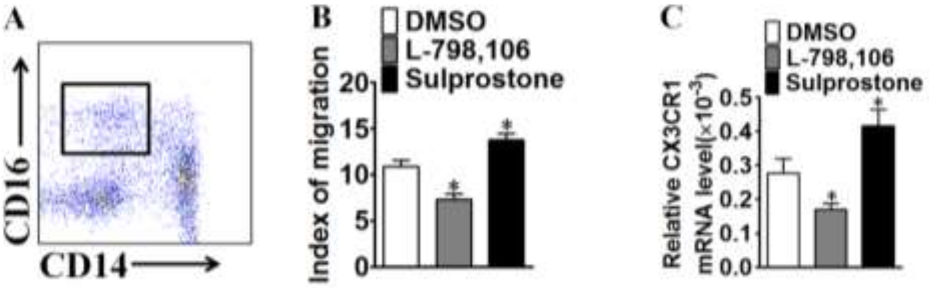

Supplementary Figure 16. Effect of Inhibition or activation of *Ep3* receptor on CX3CR1 expression and migration of human CD14^{dim} CD16^{+} Mos.

A. Gating strategy for CD14^{dim} CD16^{+} cells from human PBMCs. **B**. Effect of Sulprotone or L798，106 treatment on CD14^{dim} CD16^{+} Mos migration. *P < 0.05 vs. DMSO; n = 3-4. **C**. Effect of Sulprotone or L798,106 treatment on *CX3CR1* expression of CD14^{dim} CD16^{+} Mos. *P < 0.05 vs. DMSO; n = 5.

Supplementary Figure 17

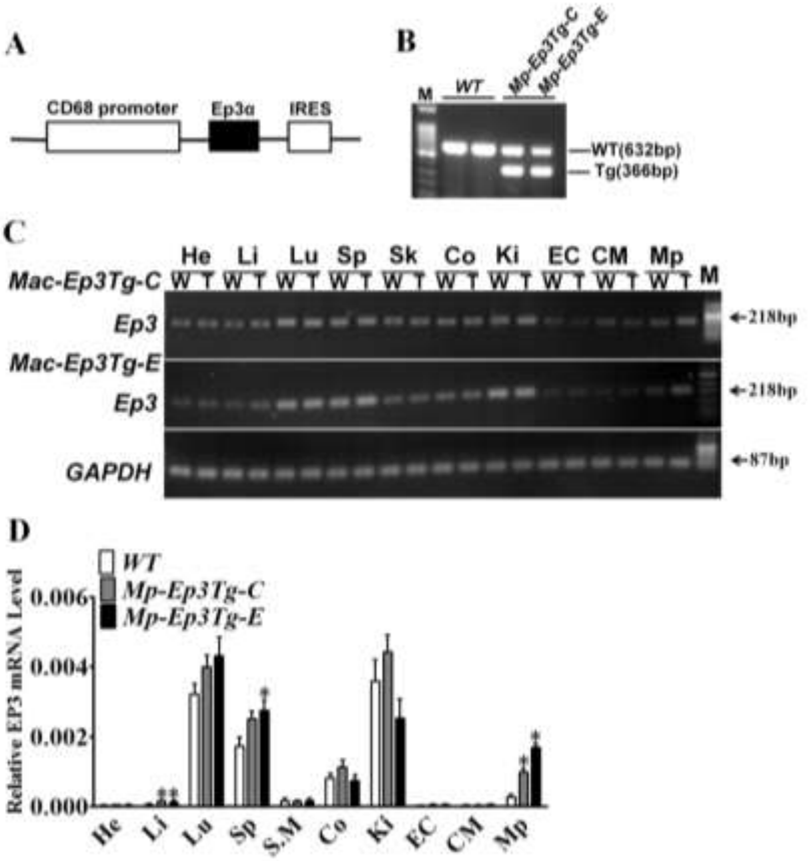

Supplementary Figure 17. Characterization of Ep3α transgenic mice

A. Transgenic construct for CD68 promoter-driven *Ep3α* expression. **B**. Genotyping of *Mac-Ep3α-Tg* mice. M, marker; TG, transgenic mice. **C.** Analysis of *Ep3* expression levels in different tissues from *Mp-Ep3Tg* mice by gel electrophoresis of RT-PCR products. He, heart; Li, liver; Lu, lung; Sp, spleen; S.M, skeletal muscle; Co, colon; Ki, kidney; EC, endothelial cell; CM, cardiomyocyte cell; Mp, macrophage. W, WT mice; T, transgenic mice. **D**. Relative *Ep3* mRNA expression levels in different tissues from *Mp-Ep3Tg* mice. **P < 0.05 vs. wild-type (WT), n = 6.

Supplementary Figure 18

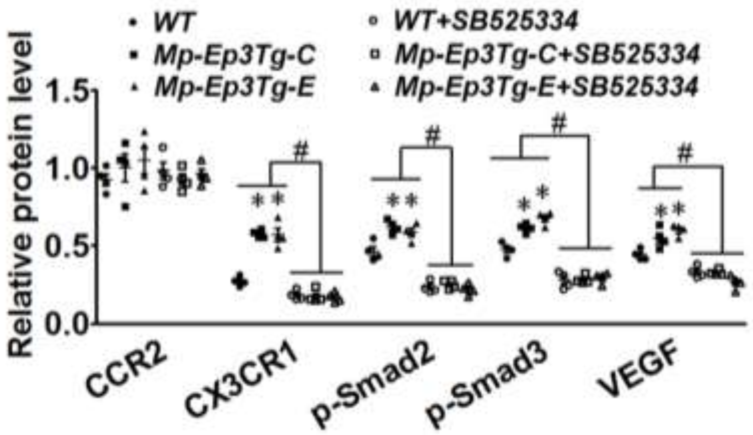

Supplementary Figure 18. Densitometric quantification of CCR2, p-Smad2/3, CX3CR1, and VEGF protein expression of peritoneal Mps after treatment from overexpression mice, as shown in Figure 5H. *P < 0.05 vs. WT, #P < 0.05 vs. Control, n = 4.

Supplementary Figure 19

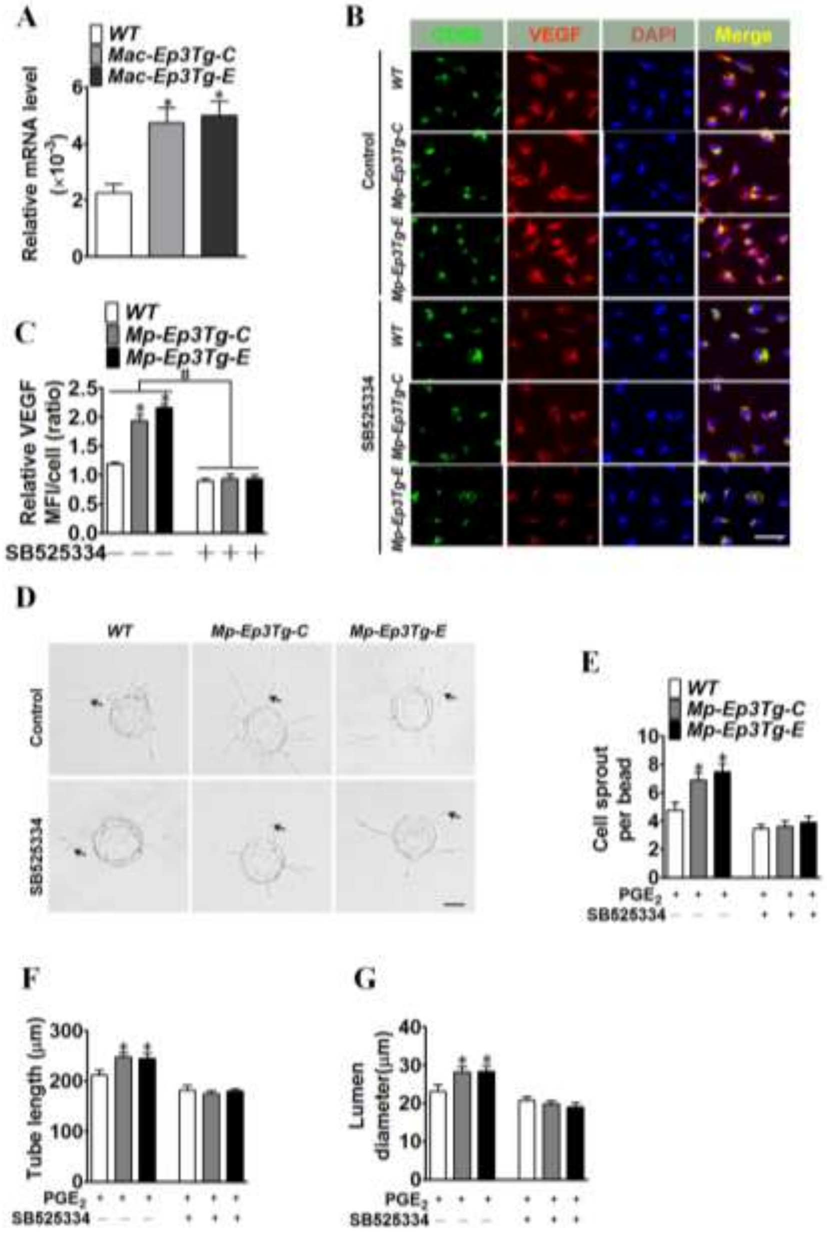

Supplementary Figure 19. Mp-Ep3 overexpression enhances VEGF secretion and tube formation of co-cultured HUVECs *in vitro*.

A.*VEGF* mRNA expression level in peritoneal Mos/Mps from *WT, Mp-Ep3Tg-C, and Mp-Ep3Tg-E m*ice. *P < 0.05 vs. WT, n =10. **B.** Representative immunostaining of CD68 (green) and VEGF (red) in peritoneal Mps from *WT, Mp-Ep3Tg-C, and Mp-Ep3Tg-E m*ice mice. Scale bar, 20 μm. **C**. Quantitation of VEGF signaling in $CD68^+$ cells in Mos/MPs, as shown in (**B**). *P < 0.05 vs. WT, #P < 0.01 as indicated, n = 6-7. **D-G.** Fibrin bead-bound HUVECs were co-cultured with Mps from *WT, Mp-Ep3Tg-C,* and *Mp-Ep3Tg-E* mice, and multiple capillary-like sprouts were displayed at day 7 (**D**). Black arrow, capillary-like sprout. Scale bar, 20 μm. The number of vessel sprouts per bead (**E**), the lumen diameter (**F**), and single-tube length per bead (**G**) were quantified at day 7. *P < 0.05 vs. WT, n = 4.

Supplementary Figure 20

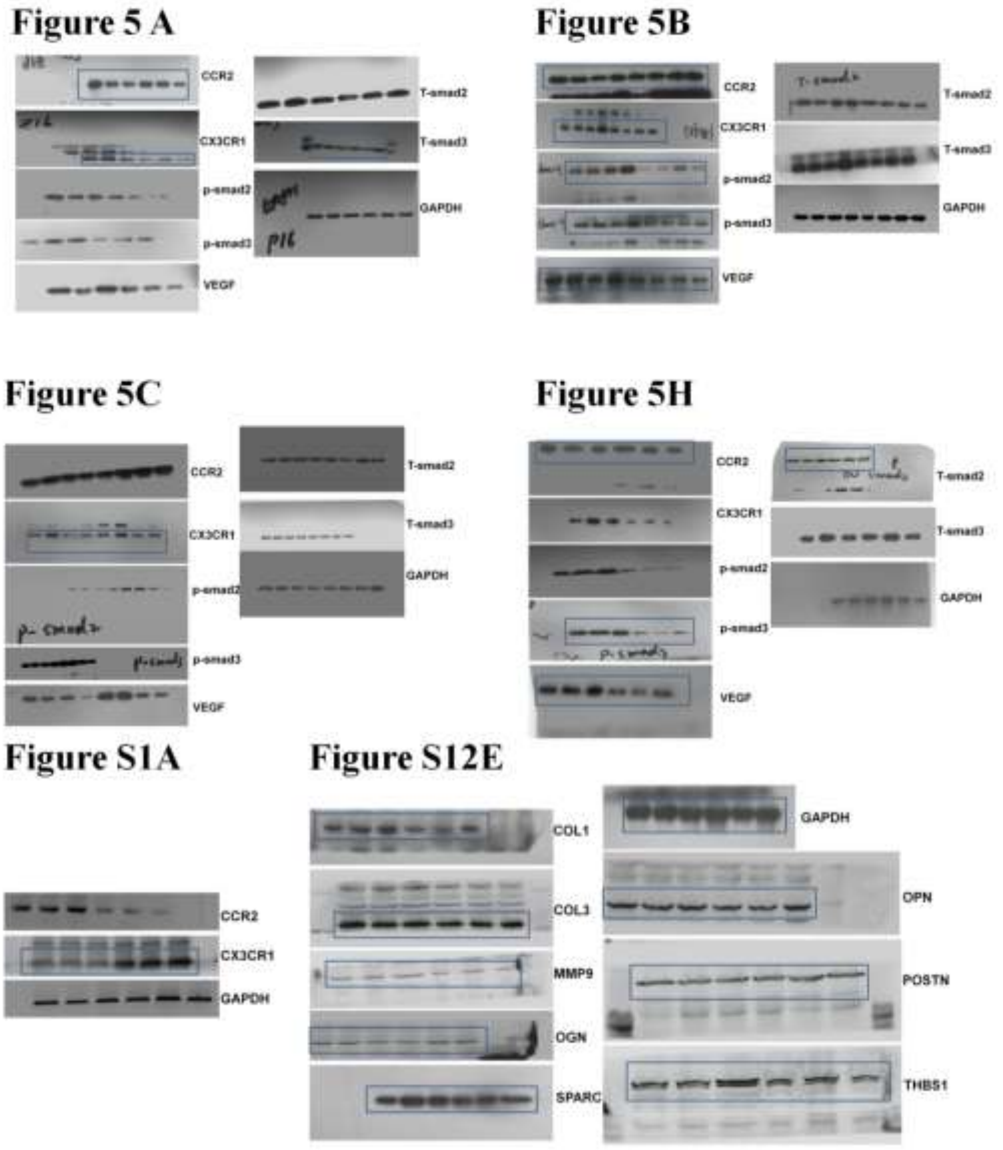

Supplementary Figure 20. The uncropped scans of western blots from Figure 5, Figure S1 and Figure S12.

Supplementary Tables

Supplementary Table 1. Effect of Mo/Mp Ep3 deletion on cardiac recovery after MI in mice

	Sham		MI	
	$Ep3^{F/F}$	$Ep3^{F/F}$;$LysM^{Cre}$	$Ep3^{F/F}$	$Ep3^{F/F}$;$LysM^{Cre}$
LVESD(mm)	3.03±0.13	2.71±0.11	4.18±0.22$^{\#}$	5.48±0.29*,$^{\#}$
LVEDD(mm)	4.22±0.10	4.02±0.08	4.86±0.15$^{\#}$	5.82±0.26*,$^{\#}$
LVESV(μl)	37.77±3.22	29.91±2.47	81.79±9.8$^{\#}$	152.53±17.96*,$^{\#}$
LVEDV(μl)	78.00±4.81	71.28±3.28	112.6±8.26$^{\#}$	173.15±17.86*,$^{\#}$
FS(%）	28.45±1.92	32.45±2.38	14.53±2.23$^{\#}$	6.21±1.01*,$^{\#}$
SV(%)	43.07±1.94	43.08±2.77	30.81±3.21$^{\#}$	20.61±1.53*,$^{\#}$
CO(ml/min)	17.02±1.45	17.43±1.97	11.96±1.73$^{\#}$	9.21±1.30*,$^{\#}$
HR(BPM)	391.81±7.93	401.3±6.99	389.75±6.38	405.18±6.87
BW (g)	25.22±1.03	25.47±2.21	25.69±1.09	26.36±1.59
LVW/BW(mg/g)	3.22±0.04	3.13±0.06	4.43±0.03	4.19±0.01
HW/BW(mg/g)	4.65±0.10	4.37±0.08	6.17±0.24$^{\#}$	6.77±0.44*,$^{\#}$
LW/BW(mg/g)	4.25±0.08	4.34±0.09	6.31±0.23$^{\#}$	7.34±0.42*$^{\#}$

LVESD, Left ventricular end-systolic dimension; LVEDD, Left ventricular end-diastolic dimension; LVESV, Left ventricular end-systolic volume; LVEDV, Left ventricular end-diastolic volume; FS, Fractional Shortening; SV, Stroke volume; CO, Cardiac output; HR, Heart rate. B W, Body weight; LVW, Left ventricular weight; HW, Heart weight; LW, Lung weight. *P < 0.05 vs $EP3^{F/F}$, $^{\#}$$P<0.05$ vs Sham, n=9-13.

Supplementary Table 2. Effect of Mo/Mp Ep3 deletion on cardiac function in mice challenged with dobutamine.

	Baseline		dobutamine	
	$EP3^{F/F}$	$Ep3^{F/F}$; $LysM^{Cre}$	$Ep3^{F/F}$	$Ep3^{F/F.}$’$LysM^{Cre}$
LVESD(mm)	2.65±0.21	2.62±0.11	1.33±0.20$^{\#}$	1.46±0.13$^{\#}$
LVEDD(mm)	3.52±0.27	3.63±0.19	3.38±0.13$^{\#}$	3.31±0.67$^{\#}$
LVESV(μl)	33.35±4.84	27.84±2.04	12.58±0.67$^{\#}$	12.89±17.96$^{\#}$
LVEDV(μl)	64.44±4.49	63.86±4.13	47.22±2.57$^{\#}$	45.13±3.42$^{\#}$
EF(%)	64.23±5.60	65.64±2.67	87.51±3.40$^{\#}$	85.40±2.13$^{\#}$
FS(%）	35.15±4.17	35.60±1.96	58.51±5.15$^{\#}$	54.25±2.65$^{\#}$
SV(%)	32.53±3.89	37.11±3.15	34.09±2.97	35.73±2.36
CO(ml/min)	14.22±1.72	15.78±1.36	18.17±1.13$^{\#}$	17.25±1.15$^{\#}$
HR(BPM)	401.79±7.14	400±10.81	479.94±4.94$^{\#}$	487.13±7.29$^{\#}$

$^{\#}P < 0.05$ vs baseline. n=6-7.

Supplementary Table 3. Histological analysis of infarcted hearts from EP3$^{F/F}$Lys^{Cre} and EP3$^{F/F}$ mice at day 14 after MI.

	EP3$^{F/F}$（n）	Ep3$^{F/F}$;Lys^{Cre} (n)
Total Mo/Mps (×10^4/heart)	9.45±0.91(6)	5.67±0.51*(6)
Ly6C^{high} (×10^4/heart)	1.22±0.19(6)	0.89±0.12(6)
Ly6C^{low} (×10^4/heart)	8.23±0.51(6)	4.79±0.58*(6)
Angiogenesis (CD31$^+$PCNA$^+$ cells/field)	15.52±1.53(7)	7.01±0.49*(7)
Fibrosis (Collagen % scar area)	53.46±1.94(6)	40.51±0.57*(6)
Myofibroblast (SMA$^+$ area %)	9.68±0.36(6)	5.92±0.40*(6)
Infarct area(%)	29.91±1.81(5)	44.87±3.52*(5)
EF(%)	30.00±4.19(13)	14.18±2.30*(11)

* P<0.05 vs EP3$^{F/F}$.

Supplementary Table 4. Effect of BTM on cardiac function of Ep3 KO mice after MI

	WT→WT	WT→KO	KO→WT	KO→KO
LVESD(mm)	3.98±0.12	4.01±0.1	4.99±0.42*	4.91±0.28*,#
LVEDD(mm)	4.55±0.1	4.79±0.13	5.32±0.37*	5.29±0.16*,#
LVESV(μl)	70.88±5.19	81.24±4.51	122.84±14.96*	122.81±9.40*,#
LVEDV(μl)	95.61±5.55	108.16±6.48	141.43±16.86*	138.8±7.35*,#
FS(%）	12.58±1.43	11.36±0.88	6.14±0.64*	6.72±0.69*,#
SV(%)	25.60±2.98	26.91±2.94	18.59±2.56*	15.88±0.85*,#
CO(ml/min)	10.88±1.06	10.86±1.47	7.90±1.28*	6.46±0.41*,#
HR(BPM)	408.50±13.45	428±10.03	419±6.76	396±3.03

*$P < 0.05$ vs WT → WT mice, #$P<0.05$ vs WT → KO mice, n=8-11.

Supplementary Table 5. Effect of TGFβ1 blocker SB525334 on cardiac function of Mp-Ep3Tg mice after MI.

	Control			SB525334		
	WT	Mp-EP3Tg-C	Mp-EP3Tg-E	WT	Mp-EP3Tg-C	Mp-EP3Tg-E
LVESD(mm)	4.41±0.22	3.63±0.38*	3.23±0.23*	4.65±0.49*$^{,\#}$	5.13±0.23*$^{,\#}$	5.02±0.32*$^{,\#}$
LVEDD(mm)	4.95±0.29	4.50±0.29*	4.07±0.17*	4.98±0.47*$^{,\#}$	5.52±0.21*$^{,\#}$	5.34±0.27*$^{,\#}$
LVESV(μl)	94.18±10.27	62.37±13.65*	43.40±6.06*	109.35±28.35*$^{,\#}$	128.8±12.23*$^{,\#}$	123.61±17.43*$^{,\#}$
LVEDV(μl)	119.3±9.03	96.62±14.71*	74.08±6.70*	125.89±29.31*$^{,\#}$	151.8±12.74*$^{,\#}$	140.89±15.83*$^{,\#}$
FS(%)	11.69±1.54	20.82±4.41*	21.33±2.57*	7.11±1.29*$^{,\#}$	7.4±0.81*$^{,\#}$	6.41±1.33*$^{,\#}$
SV(%)	25.10±2.04	34.25±3.03*	30.67±1.04	16.55±2.02*$^{,\#}$	22.09±1.72*$^{,\#}$	17.29±2.08*$^{,\#}$
CO(ml/min)	11.34±1.83	14.52±2.64*	16.35±2.45*	7.78±2.34$^{\#}$	9.09±1.18$^{\#}$	8.11±2.47$^{\#}$
HR(BPM)	484.4±33.52	441.2±14.11	453.24±19.5	466.22±19.18	458.1±18.32	469.43±19.58

*P < 0.05 vs WT mice, $^{\#}P$ < 0.01vs Control, n=7-8.

Supplementary Table 6. Primers for real-time PCR analysis in mice

Gene	Sense	Anti-sense
CCR2	ATCCACGGCATACTATCAACAT	CCAAGGCTCACCATCATCGTAG
CX3CR1	CCTGTTATTTGGGCGACATT	ACGCCCAGACTAATGGTGAC
EP1	TAACGATGGTCACGCGATGG	ATGCAGTAGTGGGCTTAGGG
EP2	GCTCGCCTGCAACATCAGCGTTA	AGCTCGGAGGTCCCACTTTTCCT
EP3	CGCACAGCAACCTGTCAAGTA	CCCCACTAAGTCGGTGGAGC
EP4	GGATCATGTGTGTGGTGTCC	GCAGAACTTCCGAAGAAGGA
IL1	TCTCAACCAATCAGCACACCCGA	GATGTAGCGGAGGCTAGAGTTGC
IL12	ATGGCCATGTGGGAGCTGGAGAAA	GGTGGAGCAGCAGATGTGAGTGGCT
IL-13	CCTGGCTCTTGCTTGCCTTGG	TCTTGTGTGATGTTGCTCA
MMP-9	TTTGAGTCCGGCAGACAATCC	CAACCGTCCTTGAAGAAATG
TNFα	TGCCTATGTCTCAGCCTCTTCG	AGGCCATTTGGGAACTTCT
TGFβ	TGTTAAAACTGGCATCTGA	GTCTCTTAGGAAGTAGGT
VEGF	GCACATAGAGAGAATGAGCTT	CCCTCCGCTCTGAACAAGGCT
GAPDH	CCCTTATTGACCTCAACTACA	TGGTGAGGGGCCATCCACAGTCTTCTG

Supplementary Table 7. Primers for Chip PCR analysis

Gene	Sense	Anti-sense
S1	AGGAAGCGGATAGAATGCAG	CATCTACCCATGTGCCAGAG
S2	ACACGGATGAGATTGGTTGA	TTGCCATCTGAGCTGTATCC
S3	TTGAAATGCAGGCTCTCTTG	GGTTTCCTGTGTGGTACCCT
S4	GGTCAATCAGGCACAGTCAC	TCAAGTTACGAGCGTGCAA
S1′	GCC AGA CTA CAC AGTGCA TA	GCT TAT CTG AGC CCT TGT CTG
S2′	GGATCATGTGTGTGGTGTCC	GCAGAACTTCCGAAGAAGGA
hS	TAAGTGGCACCTCTCTCCCT	TCTTTAGATGCTGCCACAGG
h S1′	GCATTCCCATTCTCAGTCC	GAAGAGTGGGACCAGTCAGT
h S2′	CTGGGTGGATAATCAGACTG	GCTGGTTTCTGACCTGGCTA
hS3′	ATGAGTCTGGGCTTGGGCTGATAG	TGATGATTCAAACCTACCCGCC
h S4′	CTCAGTTCCCTGGCAACATCTG	TGGGAGGGAAGAGGACCTGTT
h S5′	AGCCCATTCCCTCTTTAGCCAG	ACACTCACTCACCCACACAGACAC

Supplementary Table 8. Primers for plasmids construction

Gene	Sense	Anti-sense
S3+S4	CGTTGAGCTTGCTTGTCACAGC	CGATACAAGTTTGAGACCAGCCTAG
S1 '+ S2 '	GTTTAGAAGATGAACCGTAAGCCTAGGC	TCTCTCTGTTCGCTCGCCAGCGCGCT
hS	GCCTGTTTGCTTACGTGCAGAC	GGATACTCAGGCTCAGGTTTGTGT
hS3 '-hS5 '	ACAGTGACCGTGGAGACTCAAG	GAACAAAGCTGAACCTAAGGATCT
